# Selective targeting of IL-2 to NKG2D bearing cells for improved immunotherapy

**DOI:** 10.1038/ncomms12878

**Published:** 2016-09-21

**Authors:** Reza Ghasemi, Eric Lazear, Xiaoli Wang, Saeed Arefanian, Alexander Zheleznyak, Beatriz M. Carreno, Ryuji Higashikubo, Andrew E. Gelman, Daniel Kreisel, Daved H. Fremont, Alexander Sasha Krupnick

**Affiliations:** 1 Department of Surgery, Washington University in St Louis, 660 South Euclid Avenue, St Louis, Missouri 63110, USA; 2 Department of Pathology & Immunology, Washington University in St Louis, 660 South Euclid Avenue, St Louis, Missouri 63110, USA; 3 Department of Medicine, Washington University in St Louis, St Louis, Missouri 63110, USA; 4 Department of Molecular Microbiology, and Biochemistry & Molecular Biophysics, 660 South Euclid Avenue, St Louis, Missouri 63110, USA; 5 The Alvin Siteman Cancer Center of Washington University School of Medicine, 4921 Parkview Place, St Louis, Missouri 63110, USA

## Abstract

Despite over 20 years of clinical use, IL-2 has not fulfilled expectations as a safe and effective form of tumour immunotherapy. Expression of the high affinity IL-2Rα chain on regulatory T cells mitigates the anti-tumour immune response and its expression on vascular endothelium is responsible for life threatening complications such as diffuse capillary leak and pulmonary oedema. Here we describe the development of a recombinant fusion protein comprised of a cowpox virus encoded NKG2D binding protein (OMCP) and a mutated form of IL-2 with poor affinity for IL-2Rα. This fusion protein (OMCP-mutIL-2) potently and selectively activates IL-2 signalling only on NKG2D-bearing cells, such as natural killer (NK) cells, without broadly activating IL-2Rα-bearing cells. OMCP-mutIL-2 provides superior tumour control in several mouse models of malignancy and is not limited by mouse strain-specific variability of NK function. In addition, OMCP-mutIL-2 lacks the toxicity and vascular complications associated with parental wild-type IL-2.

## Abstract (web summary)

High-affinity IL-2Rα expressed by T_regs_ mitigates the potential of IL-2 use in cancer therapy. Here, the authors fuse IL-2 with an NKDG2 binding domain, and show that it induces IL-2 signalling selectively in NKG2D^+^ cells, delaying tumour growth in mice without the side effects of conventional IL-2 therapy.

## Graphical abstract

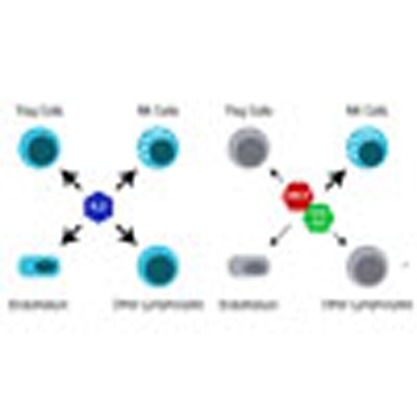

The IL-2Rα chain serves to capture IL-2 at the cell surface to facilitate subsequent binding to the signalling part of the receptor, namely the IL-2Rβγ chains. Resting cytotoxic lymphocytes, such as natural killer (NK) and CD8^+^ T cells, are believed to express little to no IL-2Rα at the cell surface and are thus not activated by low-dose IL-2 (ref. 1). IL-2Rα expression on these cells increases after initial activation and is required for maximum cytotoxic lymphocyte expansion^2^. High dose IL-2 can activate even resting cytotoxic lymphocytes, and is thus approved for treatment of several malignancies^3^,^4^,^5^. Most patients do not benefit from high dose IL-2 therapy, however, due to activation of regulatory T cell (T_regs_) and systemic complications of hemodynamic instability, generalized capillary leak and end organ failure due to activation of vascular endothelium^3^,^6^,^7^. Both vascular endothelium and T_regs_ express IL-2Rα and are thus preferentially activated by IL-2 over cytotoxic lymphocytes^8^. Lowering the IL-2 dose can ameliorate side effects but also decreases efficacy. Mutant forms of IL-2, such as those with substitutions of alanine for arginine at the 38 position (R38A) and/or lysine for phenylalanine at the 42 position (F42K), decrease the affinity of IL-2 for IL-2Rα and thus eliminate many side effects^9^. However, such IL-2α mutants also decrease the efficacy of immunotherapy^2^. A form of IL-2 that could preferentially activate cytotoxic lymphocytes in the absence of IL-2Rα engagement would be highly advantageous for clinical applications.

NKG2D is an activating receptor that is expressed on human NK and CD8^+^ T cells, murine NK cells and activated murine CD8^+^ T cells^10^. NKG2D recognizes MHC class-I-like stress ligands expressed on the surface of malignant or virally-transformed cells^11^. Consequently, many tumours and virally infected cells seek to counteract NKG2D-based immunity^12^,^13^. Orthopoxvirus major histocompatibility complex class I-like protein, or OMCP, is a small NKG2D binding protein secreted by monkeypox and cowpox virus infected cells. There are no OMCP related proteins encoded by current orthopoxvirus vaccine strains and thus there is very limited exposure in humans. OMCP binds both human and murine NKG2D with an affinity equal to, or greater than, all other known NKG2D ligands^14^,^15^. Therefore, OMCP could serve as an ideal targeting vector to deliver IL-2 specifically to cytotoxic lymphocytes. Here we describe the engineering of a fusion protein comprised of OMCP linked to an IL-2 variant with diminished IL-2Rα binding. This fusion construct retains the safety profile of IL-2 mutants with reduced IL-2Rα reactivity while improving NK cell expansion 10-fold compared with wild-type IL-2. Systemic administration decreases the growth and viability of both solid and liquid tumours and significantly improves animal survival. We thus describe a safe and efficacious IL-2 fusion protein that overcomes barriers associated with standard high-dose IL-2 therapy.

## Results

### OMCP-mutIL-2 activates cytotoxic lymphocytes *in vitro*

**Figure 1:**
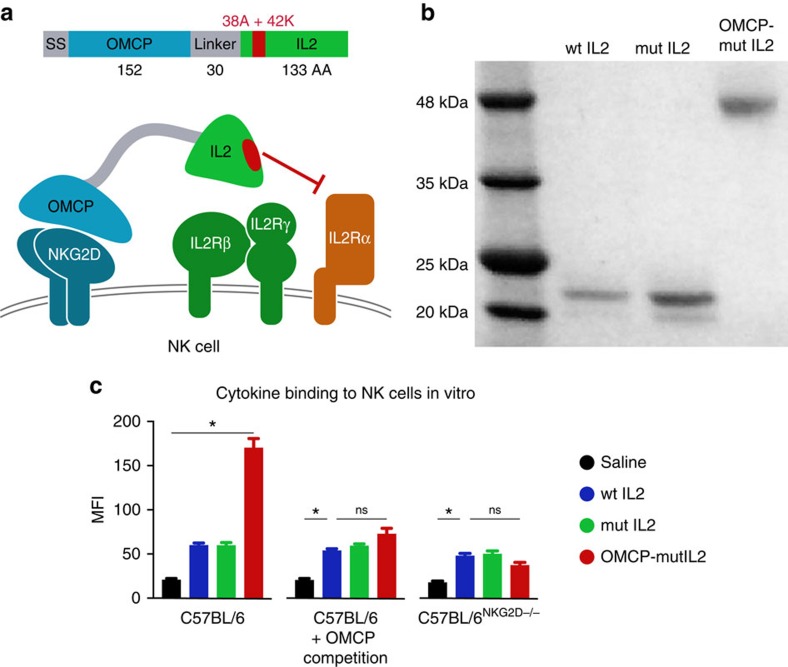
Generation of OMCP-mutIL-2. (**a**) Schematic structure of OMCP-mutIL-2. (**b**) Molecular mass of OMCP-mutIL-2 compared with mutIL-2 and wild-type IL-2 by Coomassie staining. wt IL-2, mutIL-2 and OMCP-mutIL-2 were produced in FreeStyle 293-F cells and purified from supernatants by Ni-NTA chromatography. The proteins have higher molecular mass due to glycosylation. The lower migrating band for mutIL-2 corresponds to unglycosylated protein. Based on differences in molecular mass all cytokines and construct were administered on a molar basis with 1 μl of 4.4 μM solution defined as 1000 IU equivalents (IUe) throughout the manuscript. This effectively allows for equimolar comparison between wt IL-2, mutIL-2 and OMCP-mutIL-2 despite different molecular mass. (**c**) Binding of various biotin-labelled cytokines and constructs to wild-type C57BL/6 splenic NK cells without (left panel) or with (middle panel) preincubation with OMCP. Binding of biotin-labelled cytokines and constructs to C57BL/6^NKG2D−/−^ NK cells (right panel). As described in the methods spleen cells were co-cultured with equimolar concentration of biotinylated cytokines and fusion construct at 4 °C followed by labelling with phycoerythrin (PE) conjugated streptavidin and mixed fluorochrome-conjugated antibodies for defining of NK cells. Data were acquired flow cytometrically and is representative of three separate experiments with MFI comparison of PE-labelled constructs on NK cells (viable, NK1.1+, CD3−, CD19−). Data analysis performed by ANOVA for multiple comparisons and unpaired *t*-test for individual comparisons and shown as mean±s.e.m. All bar graphs represent mean±s.e.m. ns *P*>0.05; *P<0.05; black=saline; blue=wtIL-2, red=OMCP-mutIL-2, green=mutIL-2.

We designed an IL-2 fusion protein combining the high affinity NKG2D ligand OMCP with an IL-2 mutated to reduce IL-2Rα reactivity (mutIL-2). Our construct, termed OMCP-mutIL-2, consists of the 152 residue OMCP protein fused to the N-terminus of the 133 amino acid R38A/F42K mutant form of human IL-2 (mutIL-2) via a flexible 30 residue linker (Fig. 1a,b). We incubated equimolar concentration of biotinylated wild-type IL-2 (wtIL-2), mutIL-2 or OMCP-mutIL-2 with C57BL/6 splenocytes at 4 °C *in vitro* and compared binding flow cytometrically. The addition of the OMCP to mutIL-2 increased the retention of the fusion protein to NK cells compared with mutIL-2 or wtIL-2 (Fig. 1c left panel) as evidenced by significantly higher MFI. This increase in lymphocyte binding depended on functional and reactive NKG2D, as competitive preincubation of splenocytes with free monomeric OMCP eliminated enhanced binding of OMCP-mutIL-2 to NK cells (Fig. 1c middle panel). Consistent with this no increase in OMCP-mutIL-2 binding over mutIL-2 was evident in NK cells from C57BL/6^NKG2D−/−^ mice (Fig. 1c right panel). No increased binding of OMCP-mutIL-2 over mutIL-2 was evident for either wild-type or C57BL/6^NKG2D−/−^ B or T lymphocytes (Supplementary Fig. 1). Taken together our data demonstrate that a fusion protein consisting of a cytokine and an NKG2D ligand may have utility for targeting NKG2D expressing lymphocytes such as NK cells.

**Figure 2:**
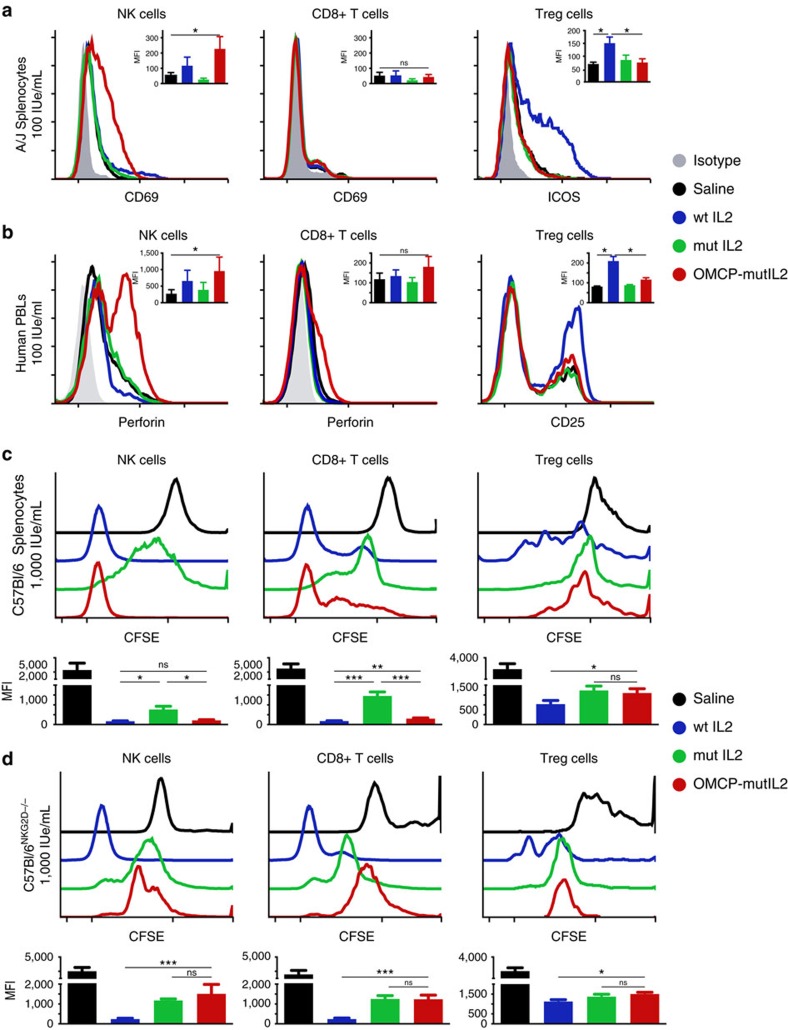
*In vitro* evaluation of OMCP-mutIL-2. (**a**) *In vitro* flow cytometrically evaluated activation of A/J spleen-derived lymphocyte subsets after 36 h of culture in 100 IUe ml^−1^ of cytokines or OMCP-mutIL-2 construct. Graph demonstrating one representative histogram plot and median fluorescent intensity (MFI) ±s.e.m. across 5–7 separate experiments, containing one to two mice per group per experiment, as a graph in right upper corner. (**b**) *In vitro* activation of human peripheral blood lymphocyte subsets after 36 h of culture in 100 IUe ml^−1^ of cytokines or OMCP-mutIL-2 construct. Graph demonstrating one representative histogram plot and MFI±s.e.m. across 4–5 separate experiments as graph in right upper corner. Proliferation of C57BL/6 wild-type (**c**) or C57BL/6^NKG2D−/−^ (**d**) splenic lymphocyte subsets after 5-day culture in 1,000 IUe ml^−1^ of cytokines or OMCP-mutIL-2. Graph demonstrating one representative histogram plot of four separate experiments. Composite MFI±s.e.m. shown as graph below the CFSE histogram plots. Comparison of MFI performed by ANOVA for multiple comparisons and unpaired *t*-test for each individual group. ns *P*>0.05; *P<0.05; **P<0.01; ***P<0.001; black=saline; blue=wtIL-2, red=OMCP-mutIL-2, green=mutIL-2.

Based on this data we next set out to examine the efficacy of OMCP-mutIL-2 in activation of NK cells from two different strains of mice (A/J and C57BL/6) with poor and robust NK function, respectively^16^. Compared with wtIL-2 or mutIL-2, OMCP-mutIL-2 strongly upregulated CD69 on NK cells of both strains after a two-day co-culture with 100 IUe ml^−1^ of cytokine (Fig. 2a left panel; Supplementary Fig. 2a). Hundred fold higher concentrations of wtIL-2 or mutIL-2 induced similar increases in CD69 expression compared with OMCP-mutIL-2 (Supplementary Fig. 2b). CD8^+^ T cells demonstrated no upregulation of CD69 (Fig. 2a middle panel). This is consistent with the low surface expression of IL-2 receptors and NKG2D on unactivated, resting murine T lymphocytes. Activation of CD4^+^Foxp3^+^ T_regs_, as measured by upregulation of ICOS, was evident after co-culture with wtIL-2, but not with mutIL-2 nor OMCP-mutIL-2 (Fig. 2a right panel). While CD69 upregulation is only a transient marker of lymphocyte activation, acquisition of cytotoxic mediators such as perforin has been described as a reliable measure of cytotoxicity^17^. To expand our murine observations we co-cultured freshly isolated human peripheral blood lymphocytes (PBLs) with 100 IUe ml^−1^ of OMCP-mutIL-2, wtIL-2 or mutIL-2 in a similar manner to murine splenocytes and evaluated intracellular perforin accumulation flow cytometrically. Consistent with murine CD69 data, human NK cells treated with OMCP-mutIL-2 had higher perforin levels than those treated with wild-type IL-2, mutIL-2 or saline (Fig. 2b left panel). Thus, exposure to OMCP-mutIL-2 results in preferential activation of human NK cells as well. Limited activation of CD8^+^ T cells was evident with any of the constructs (Fig. 2b middle panel) and human T_regs_ were preferentially activated by wtIL-2 but not mutIL-2 or OMCP-mutIL-2 similar to mice (Fig. 2b right panel).

Significant proliferation of both C57BL/6 NK and CD8^+^ T cells was evident *in vitro* after incubation with 1000 IUe ml^−1^ of wtIL-2 and OMCP-mutIL-2 but not mutIL-2 (Fig. 2c left and middle panel) whereas CD4^+^Foxp3^+^ T_regs_ proliferated only with wtIL-2 (Fig. 2c right panel). Interestingly, in human peripheral blood mononuclear cells (PBMC), derived from CMV-seropositive donors, *in vitro* stimulation with a CMV pp65 peptide in the presence of 100 IUe ml^−1^ wtIL-2 and OMCP-mutIL-2 led to antigen (pp65)-specific CD8^+^ T cells expansion (Supplementary Fig. 2c). Furthermore OMCP-mutIL-2 activation of C57BL/6 CD8^+^ T cells and NK cells was equivalent to mutIL-2 in NKG2D^−/−^ splenocytes, supporting the importance of NKG2D targeting for the superior effectiveness of OMCP-mutIL-2 (Fig. 2d; Supplementary Fig. 2d). Taken together these data indicate that exposure to OMCP-mutIL-2 results in preferential NK activation *in vitro*, that is superior or equivalent to wtIL-2 in a dose-dependent manner without concomitant T_reg_ activation. While CD8^+^ T cells require longer exposure to higher doses for activation, or concomitant T cell receptor stimulation with a relevant peptide, OMCP-mutIL-2 is able to activate them at a comparable level to wtIL-2.

### OMCP-mutIL-2 offers a favourable safety profile *in vivo*

**Figure 3:**
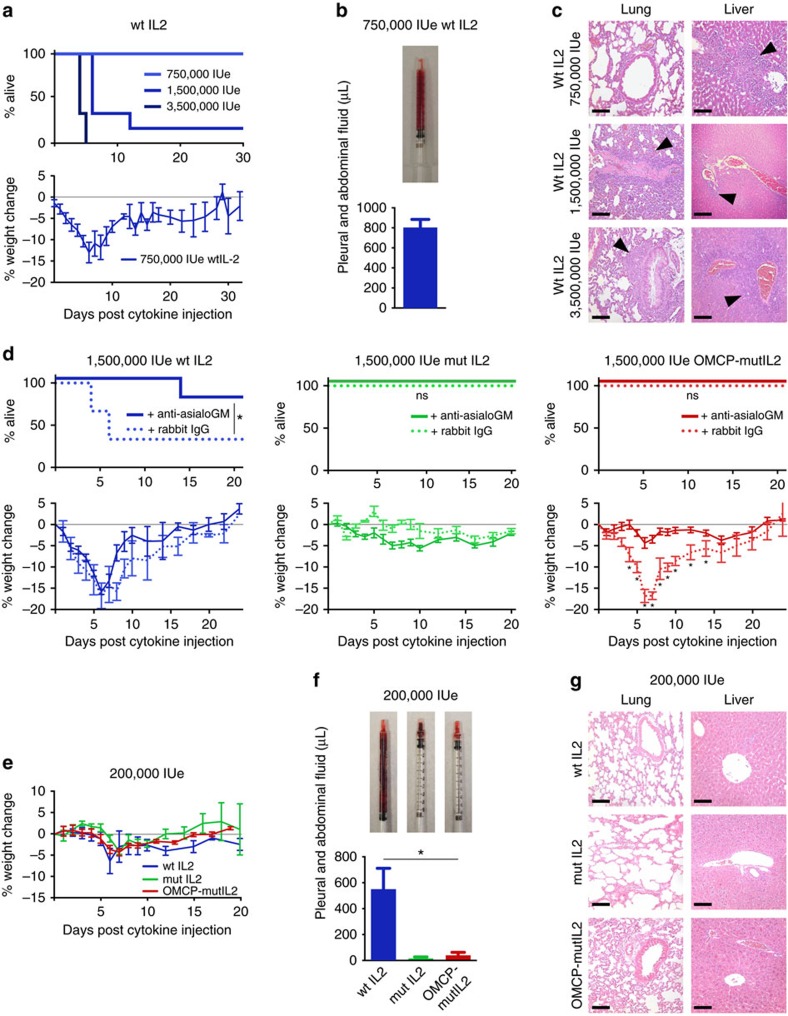
*In vivo* dosing of different forms of IL-2 in A/J Mice. (**a**) Animal mortality and morbidity, as assessed by survival (top) and weight loss (bottom); (**b**) accumulation of ascites and pleural fluid (representative syringe; top; average from all mice in the group; bottom) and (**c**) tissue inflammation after administration of wtIL-2 (arrows point to inflammatory infiltrates). All H+E figures presented at 200 × exposure with scale bar at bottom left=100 μm. (**d**) Animal mortality (top) and morbidity as assessed by weight loss (bottom) after administration of high dose wtIL-2 (left), OMCP-mutIL-2 (middle) and mutIL-2 (right) in anti-AsialoGM1 (solid line) or rabbit IgG-treated (dotted line) A/J mice. Weight loss compared by unpaired *t*-test at each individual day between NK sufficient and depleted mice. Kaplan–Meier survival graphs compared by Log-rank (Mantel–Cox) test. (**e**) Weight loss, (**f**) ascites (representative syringe-top; average from all mice in the group-bottom) and (**g**) organ inflammation in mice treated with 200,000 IUe of either wtIL-2, OMCP-mutIL-2 or mutIL-2. Comparison of ascites done by unpaired *t*-test between wt IL-2 and mutIL-2 and OMCP-mutIL-2 treated mice respectively. All H+E figures presented at 200 × exposure with scale bar at bottom left=100 μm. All graphs represent 4–6 A/J mice per treatment condition performed in at least two separate experiments. ns *P*>0.05; *P<0.05; black=saline; blue=wtIL-2, red=OMCP-mutIL-2, green=mutIL-2.

Dose-dependent toxicity can limit cytokine administration *in vivo*^18^. While A/J mice tolerated 750,000 IUe of wtIL-2, mortality was evident at higher doses (Fig. 3a). Even at the 750,000 IUe dose, mice demonstrated distress, weight loss, decreased food consumption, ascites and hepatic inflammation (Fig. 3a–c and Supplementary Fig. 3a). These side-effects resemble the capillary leakage and clinical distress associated with high dose IL-2 therapy in humans^7^. Depletion of NK cells through the administration of anti-Asialo-GM1 ameliorated mortality, but not weight loss, induced by high dose wtIL-2 (1,500,000 IUe) in A/J mice. This confirmed that side effects of wtIL-2 can occur independent of NK cells (Fig. 3d). Unlike with wtIL-2 no animal death was evident after high dose OMCP-mutIL-2 or mutIL-2 treatment in the presence or absence of NK cells. Animal weight loss after administration of high dose OMCP-mutIL-2 occurred only in NK cell-sufficient mice, suggesting that toxicity of this fusion protein was solely due to immunoactivation (Fig. 3d). A regimen of low-dose, 200,000 IUe of wtIL-2, mutIL-2 or OMCP-mutIL-2, was well tolerated in A/J mice with minimal weight loss, distress or organ inflammation (Fig. 3e–g). Capillary leak, however, was still evident at this dosing regimen for wtIL-2 (Fig. 3f). C57BL/6 mice were able to tolerate higher doses of wtIL-2 but still suffered significant morbidity when administered a regimen equivalent to or higher than 750,000 IUe (Supplementary Fig. 3b). Interestingly weight loss was ameliorated in C57BL/6^NKG2D−/−^ mice treated with OMCP-mutIL-2, consistent with the importance of NKG2D for immunoactivation by our fusion protein (Supplementary Fig. 3c). Taken together these data show that OMCP-mutIL-2 offers a safety advantage compared with wtIL-2.

### OMCP-mutIL-2 expands and activates NK Cells *in vivo*

**Figure 4:**
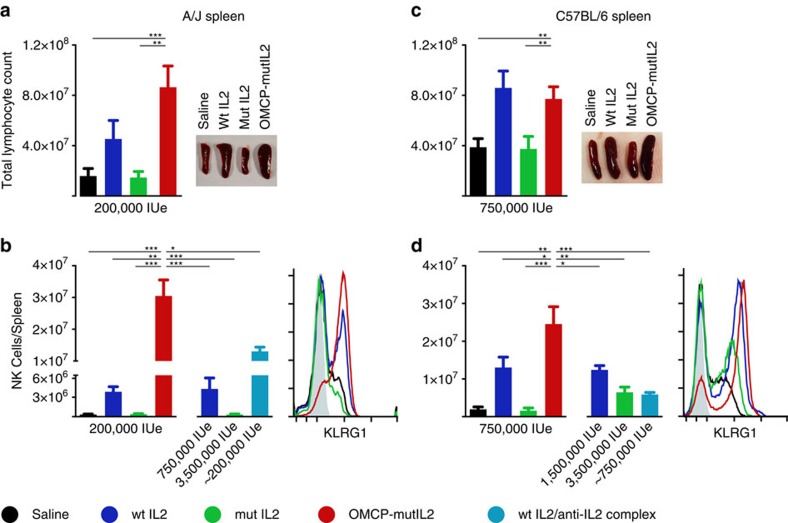
Immunoactivation with IL-2 and construct administration *in vivo*. (**a**) Total splenocyte counts in AJ mice after a five-day course of 200,000 IUe of wtIL-2 (blue), mutIL-2 (green) and OMCP-mutIL-2 (red). (**b**) NK cell expansion and activation after wtIL-2, mutIL-2, OMCP-mutIL-2, high dose wtIL-2, high dose mutIL-2 and wtIL-2/anti-IL-2 complexes measured by cell counts in the spleen (top) and KLRG1 upregulation (bottom). Expansion of splenocytes (**c**) and NK cells (**d**) in C57BL/6 mice treated with 750,000 IUe of cytokine or construct. All graphs represent an average cell count ± s.e.m. from 5–10 mice per group performed as 4–7 separate experiments for each strain of mice. Comparison performed by unpaired *t*-test between groups as indicated by the lines. ns *P*>0.05; *P<0.05; **P<0.01; ***P<0.001; black=saline; blue=wtIL-2, red=OMCP-mutIL-2, green=mutIL-2, turkoise=wtIL-2 complexed to anti-IL-2.

When A/J mice received a regimen of 200,000 IUe of cytokine or fusion protein, given as 10 equal doses over 5 days, both wtIL-2 and OMCP-mutIL-2 increased splenocyte numbers compared with saline-treated controls (Fig. 4a). OMCP-mutIL-2 led to a substantial expansion and activation of NK cells as measured by cell number and surface KLRG1 levels (Fig. 4b). Remarkably, in OMCP-mutIL-2 treated mice, NK cells comprised close to half of all splenic lymphocytes, paralleling or even surpassing the total lymphocyte counts of saline or mutIL-2-treated mice (Fig. 4a versus b). NK cell expansion by 200,000 IUe of OMCP-mutIL-2 was greater than that seen with near toxic doses of wtIL-2 (750,000 IU), high dose mutIL-2 (3,500,000 IUe) or wtIL-2 complexed to anti-IL-2 antibody (clone MAB602)^19^ (Fig. 4b). Superior expansion of NK cells by OMCP-mutIL-2 was even possible at doses 2-fold lower than wtIL-2 (Supplementary Fig. 4a). Furthermore, a mutIL-2 fusion protein comprised of a different NKG2D ligand with a ∼500-fold lower affinity, ULBP3 (ref. 15), did not significantly expand NK cells but still offered superior activation compared with mutIL-2 alone (Supplementary Fig. 4a). No significant increase in CD4^+^Foxp3^−^ or CD8^+^ T lymphocytes was evident in A/J mice after wtIL-2 or OMCP-mutIL-2 treatment (Supplementary Fig. 4b). Splenic DX5^+^CD3^+^ NKT cells, while lower in number than DX5^+^CD3^−^ NK cells, did expand after OMCP-mutIL-2 treatment, albeit equivalent to wtIL-2 in the A/J strain of mice (Supplementary Fig. 4b).

In contrast to the A/J strain, C57BL/6 mice were able to tolerate higher doses of IL-2 (Supplementary Fig. 3b). At higher doses of 750,000 IUe OMCP-mutIL-2 induced greater expansion of NK cells compared with other cytokines, similar to that described for A/J mice (Fig. 4c,d). Importantly, expansion of NK cells was not evident in OMCP-mutIL-2 treated C57BL/6^NKG2D−/−^ mice, confirming the requirement for NKG2D for the function of OMCP-mutIL-2 (Supplementary Fig. 4c). Consistent with the A/J strain neither CD4^+^Foxp3^−^ nor CD8^+^ T lymphocytes expanded after treatment with OMCP-mutIL-2 but DX5^+^CD3^+^ NKT cells did expand compared with other cytokine therapy, consistent with their expression of NKG2D (Supplementary Fig. 4d). Interestingly NKG2D reactivity was still preserved in OMCP-mutIL-2-treated NK cells, as measured by degranulation to plate-bound agonistic anti-NKG2D antibody (clone A10)^20^ (Supplementary Fig. 4e). Thus, despite the fact that OMCP is an NKG2D antagonist, the concentrations needed for activation are not sufficient to significantly impede the function of the NKG2D receptor.

### OMCP-mutIL2 does not expand or activate T_regs_

**Figure 5:**
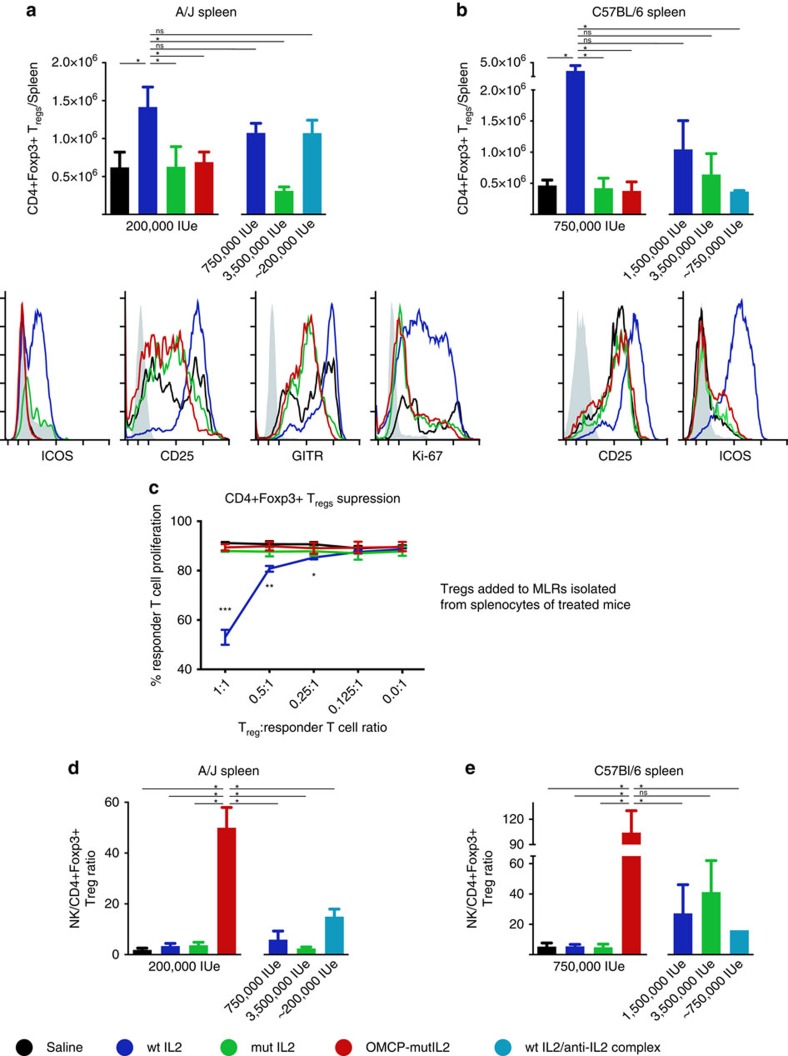
T_reg_ Activation and Expansion with IL-2 and construct administration *in vivo*. (**a**) Splenic CD4^+^Foxp3^+^ Treg expansion and activation as measured by cell counts in the spleen (top) and ICOS, CD25, GITR and KI67 expression (bottom) in A/J and C57BL/6 (**b**) mice. (**c**) *In vitro* suppression of C57BL/6^CD45.1+^ congenic T cell proliferation by CD4^+^Foxp3^+^ T_regs_ isolated from splenocytes of C57BL/6^CD45.2+Foxp3+GFP+^ mice treated with saline (black), wtIL-2 (blue), mutIL-2 (green) and OMCP-mutIL-2 (red) *in vivo*. Data representative of four separate *in vitro* experiments with comparison performed by unpaired *t*-test to saline-treated control at each ratio indicated and confirmed by ANOVA. (**d**) NK/Treg ratio in the spleen of A/J or C57BL/6 (**e**) mice treated with saline (black), wtIL-2 (blue), mutIL-2 (green) and OMCP-mutIL-2 (red) *in vivo*. All graphs in a,b,d,e represent an average cell count±s.e.m. from 5 to 10 mice per group performed as 4–7 separate experiments for each strain of mice. Comparison performed by unpaired *t*-test between groups as indicated by the lines. ns *P*>0.05; *P<0.05; **P<0.01; ***P<0.001; black=saline; blue=wtIL-2, red=OMCP-mutIL-2, green=mutIL-2; turkoise wtIL-2 complexed to anti-IL-2.

Preferential activation of IL2Rα-expressing T_regs_ has been one of the main challenges to IL-2 cytokine immunotherapy^8^. Consistent with this notion wtIL-2 led to a significant expansion and activation of CD4^+^Foxp3^+^T_regs_ as measured by total cell number and expression of ICOS, GITR, CD25 and KI-67 in A/J mice (Fig. 5a). Expansion of this cell population was also evident when wtIL-2 was complexed to anti-IL-2 antibodies (Fig. 5a)^19^. Similar results were evident for the C57BL/6 strain except for the ameliorated T_reg_ expansion when wtIL-2 was complexed to anti-IL-2 antibodies (Fig. 5b). However, for both strains of mice no expansion or activation of T_regs_ was evident after treatment with OMCP-mutIL-2 compared mutIL-2 or saline-treated controls (Fig. 5a,b).

To test functional T_reg_-mediated suppression we treated C57BL/6^CD45.2+^ mice expressing GFP on a Foxp3 promoter (kindly provided by Chyi-Song Hseih) with saline, wtIL-2, mutIL-2 or OMCP-mutIL-2 as described above. GFP-expressing T_regs_ were isolated flow cytometrically and then co-cultured with CFSE-labelled C57BL/6^CD45.1+^ congenic ‘responder' T lymphocytes, activated by soluble anti-CD3 and T cell-depleted splenocytes. Proliferation of responding T lymphocytes was measured by dilution of CFSE in CD45.1^+^CD90.2^+^ T lymphocytes. Only T_regs_ from wtIL-2-treated mice were able to inhibit T cell proliferation confirming the phenotypic data and further defining that treatment with OMCP-mutIL-2 does not result in T_reg_ activation (Fig. 5c). Furthermore, the NK/Treg ratio, which has been described as a predictive factor for success of immunotherapy^21^, was significantly increased in OMCP-mutIL-2 treated mice compared with all other conditions (Fig. 5d,e). Taken together this data suggest that treatment with OMCP-mutIL-2 offers a unique strategy for safe and efficacious NK expansion in the absence of deleterious side effects or T_reg_ activation.

### OMCP-mutIL-2 does not signal through NKG2D

**Figure 6:**
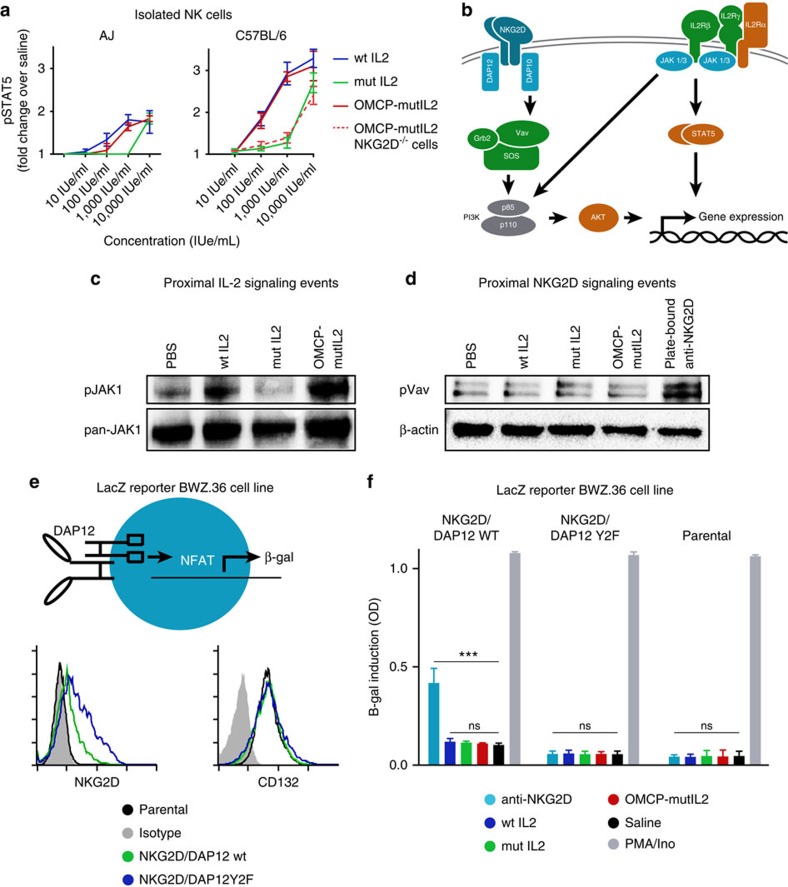
Mechanism of OMCP-mutIL-2 Signalling in NKG2D-Expressing Cells. (**a**) STAT5 phosphorylation in isolated NK cells from splenocytes of A/J (left) or C57BL/6 mice (right) by increasing doses of cytokine. (**b**) Canonical IL-2 and NKG2D signaling pathways. (**c**) Jak1 phosphorylation of Ky1.1 NK cell line *in vitro* (**d**) Vav phosphorylation in freshly isolated splenic C57BL/6 NK cells. Representative of two separate experiments with full blots demonstrated in Supplementary Fig. 7. (**e**) Construction and phenotype of BWZ.36 LacZ reporter cell line (top) and expression of NKG2D (bottom left) and CD132 (bottom right) with parental cell shown in black, functional NKG2D/DAP12 WT in green and NKG2D/mutant Y2F DAP12 in blue. Phenotype is representative of two separate experiments. (**f**) β-galactosidase expression by BWZ.36 cell line bearing functional NKG2D/DAP12 (left panel), NKG2D/mutant Y2F DAP12 (middle), or parental BWZ. 36 cell line not expressing NKG2D/DAP12. Data are representative of three separate experiments with comparison performed by ANOVA for multiple comparisons or unpaired *t*-test between individual groups as indicated by the lines above the graphs.

To evaluate IL-2 signalling we next quantitated STAT5 phosphorylation of NK cells *ex vivo*. Lower levels of STAT5 phosphorylation were evident in A/J compared with C57BL/6 NK cells at all concentrations tested (Fig. 6a) suggesting that lymphocyte dysfunction of A/J mice may partially result from inefficient IL-2 signal transduction. Interestingly in such purified NK cell preparations wtIL-2 and OMCP-mutIL-2 demonstrated identical dose-dependent STAT5 phosphorylation (Fig. 6a). In the absence of NKG2D, OMCP-mutIL-2 failed to increase STAT5 phosphorylation over mutIL-2 alone (Fig. 6a). Taken together, these data suggest that IL-2Rα engagement is important for peak IL-2 signalling even in resting NK cells, and that NKG2D-binding may substitute for IL-2Rα-interaction in IL-2-mediated signal transduction. Such data, however, opened the possibility that the superior efficacy of OMCP-mutIL-2 over mutIL-2 alone was due to concomitant signalling through NKG2D, since activation of this receptor can act as a primary or co-stimulatory factor for NK cells^20^.

Canonical dogma suggests that NKG2D signalling in resting NK cells occurs through the Dap10/Vav/Grb2 pathway^22^, while IL-2R signalling occurs through the Jak1/3/STAT5 pathway^23^(Fig. 6b). Nevertheless, alternative signalling pathways have been described^24^. To evaluate more proximal signalling events we compared Jak1 phosphorylation in the murine NKG2D expressing NK cell line Ky1.1 after incubation with wtIL-2, mutIL-2 or OMCP-mutIL-2 (ref. 25). Consistent with phospho-STAT-5 data, Jak1 phosphorylation was evident after exposure to wtIL-2 and OMCP-mutIL-2 but not saline or mutIL-2 (Fig. 6c). Since Vav controls NKG2D signalling in NK cells^22^ we next set out to evaluate phosphorylation of this signalling intermediate as a surrogate marker of NKG2D signalling. Since Ky1.1, as well as other NK cell lines such as NK92, have a high level of constitutive Vav phosphorylation (Supplementary Fig. 5) we evaluated phospo-Vav levels in freshly isolated resting splenic C57BL/6 NK cells after a 15 min exposure to cytokines. While a significant increase in phospho-Vav was evident after NKG2D signalling mediated by plate bound anti-NKG2D (clone A10), no increase in Vav phosphorylation was evident after culture with OMCP-mutIL-2, mutIL-2 or wtIL-2 (Fig. 6d). While this provided strong evidence that OMCP-mutIL-2 signals through the IL-2R, we still sought more rigorous data about the status of NKG2D signalling.

To study NKG2D signalling directly we took advantage of a reductionist approach by utilizing DAP12-NFAT driven β-galactosidase induction in BWZ.36 cells. As previously described by us and others^26^,^27^ we co-expressed NKG2D along with wild-type DAP12-GFP, or mutant non-signalling DAP12Y2F-GFP, in the BWZ.36 cells which constitutively expresses the IL2R common gamma chain (Fig. 6e). The NKG2D/DAP12 expressing cells were then co-cultured with either plate bound anti-NKG2D (clone A10), saline, wtIL-2, mutIL-2 or OMCP-mutIL-2 at 1000 IUe ml^−1^ or PMA and ionomycin for 6 h. While plate bound anti-NKG2D and PMA/ionomycin stimulation resulted in significant induction of β-galactosidase activity in wild-type DAP12 expressing cells, no such increased activity was evident in OMCP-mutIL-2 treated cultures (Fig. 6f left panel). Consistent with the requirement for NKG2D signalling no β-galactosidase induction was evident in DAP12Y2F expressing mutants or parental BWZ.36 cells after exposure to plate bound anti-NKG2D (Fig. 6f middle and right panels). Taken together these data suggest that augmented NK activation by OMCP-mutIL-2 is unlikely to be due to NKG2D activation at physiological concentrations.

### OMCP-mutIL-2 has a competitive advantage over wtIL-2

**Figure 7:**
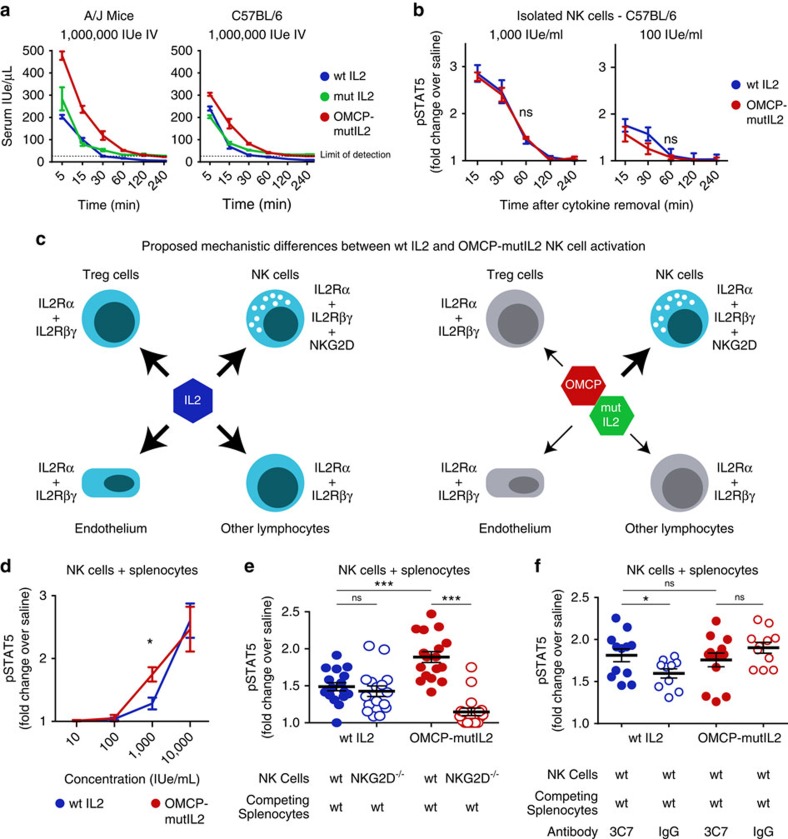
Mechanism of OMCP-mutIL-2 Competition with Stromal Cells. (**a**) Serum levels of fluorochrome-labelled cytokine or fusion protein after injection of 1 × 10^6^ IUe (i.v.) determined fluoroscopically according to a standard curve. (**b**) Decay in STAT5 phosphorylation after a 15-minute stimulation by 1,000 IUeml^−1^ (left) or 100 IUe ml^−1^ (right) of IL-2 or OMCP-mutIL-2 as determined flow cytometrically. Comparison performed by multiple unpaired *t*-tests at each individual time point. (**c**) Proposed model of competition between NK cells and stromal cells for IL-2. Width of arrow indicates proposed strength of IL-2 signaling. (**d**) Dose response in STAT5 phosphorylation of C57BL/6 NK cells in the presence of other splenocytes by wtIL-2 and OMCP-mutIL-2 as determined by flow cytometric staining. Comparison performed by multiple unpaired *t*-tests at each individual cytokine concentration. (**e**) STAT5 phosphorylation of wild-type or NKG2D^−/−^ NK cells by wtIL-2 and OMCP-mutIL-2 in the presence of competing splenocytes. (**f**) STAT5 phosphorylation, as measured by fold change over saline-treated controls, of wild-type NK cells in the presence of competing splenocytes treated with saturating concentrations of rat anti-mouse CD25 (clone 3C7) or rat IgG isotype control. Comparison performed by unpaired *t*-test between groups as indicated by the lines above the graphs. ns *P*>0.05; *P<0.05; **P<0.01; ***P<0.001; blue=wtIL-2, red=OMCP-mutIL-2, green=mutIL-2.

Antibody-IL-2 complexes improve cytokine activity by extending the duration of serum half-life^28^,^29^. To investigate whether the linking of mutIL-2 to OMCP increased serum half-life we injected 500,000 IUe of fluorescently-labelled wtIL-2, mutIL-2 or OMCP-mutIL-2 intravenously and monitored serum levels over time with periodic blood draws. Whereas OMCP-mutIL-2 had a slightly higher serum concentration at early time points, all proteins were undetectable in the blood one hour post-injection (Fig. 7a). This is shorter than the described 11–14 h serum half-life of antibody-IL-2 conjugates^28^. Despite the injection of identical amounts of cytokine, lower levels were detected in C57BL/6 than A/J mice at all time points. Strain-specific differences in the clearance of IL-2 may explain why C57BL/6 mice tolerate and require higher doses of IL-2 for NK expansion. Nevertheless it is unlikely that prolonged circulation of the fusion protein was responsible for the increased activation of NK cells by OMCP-mutIL-2 in either strain.

IL-2 signalling results in the internalization of IL-2/IL-2R, with subsequent degradation of IL-2 and IL-2Rβγ^30^,^31^. The binding of OMCP-mutIL-2 to both the IL-2 receptor and NKG2D could possibly lead to altered internalization and thus enhance NK cell activation by prolonging IL-2 signalling. To test this hypothesis, we stimulated freshly isolated NK cells for 15 min *in vitro*, replaced the culture media with cytokine-free media, and monitored STAT5 phosphorylation for four hours. Identical decay of phospho-STAT5 was evident for both wtIL-2 and OMCP-mutIL-2 (Fig. 7b). Thus, the superior activation of NK cells by OMCP-mutIL-2 could not be explained by altered duration of IL-2 signalling.

We next considered the possibility that the superior immunologic effect of OMCP-mutIL-2 over wtIL-2 *in vivo* may be the result of altered interaction with competing stromal cells (Fig. 7c). Indeed, in the presence of other splenocytes OMCP-mutIL-2 demonstrated a dose-dependent enhancement in NK STAT5 phosphorylation compared with wtIL-2 (Fig. 7d). We next explored the interplay between IL-2Rα reactivity of splenocytes and NKG2D expression on NK cells in IL-2 signal transduction. To accomplish this we isolated splenic NK cells from either wild-type or NKG2D^−/−^ C57BL/6 mice and combined them with wild-type splenocytes depleted of NK cells in a 1:20 ratio mirroring the ≈5% NK cell content in normal C57BL/6 splenocytes. Under such conditions STAT5 phosphorylation was identical between NKG2D^−/−^ and wild-type NK cells after wtIL-2 stimulation (Fig. 7e, left two columns). However, wild-type NK cells cultured with OMCP-mutIL-2 showed greater STAT5 phosphorylation relative to cultures treated with wtIL-2. Little STAT5 phosphorylation was evident in NKG2D^−/−^ NK cells treated with OMCP-mutIL-2 (Fig. 7e, right two columns). Next NK cell-depleted splenocytes were treated with saturating concentrations of IL-2Rα-blocking antibody (clone 3C7) prior to recombination with NK cells. IL-2Rα blockade led to increased STAT5 phosphorylation by wtIL-2, similar to that achieved by OMCP-mutIL-2 (Fig. 7f). Collectively, these data demonstrate that IL-2Rα expression by ‘competing' stromal cells can limit wtIL-2 mediated activation of NK cells. NKG2D-targeted, IL-2Rα-binding impaired OMCP-mutIL-2 fusion protein, however, can overcome these limitations even in the presence of competing stromal cells.

### OMCP-mutIL-2 offers superior control of malignancies *in vivo*

**Figure 8:**
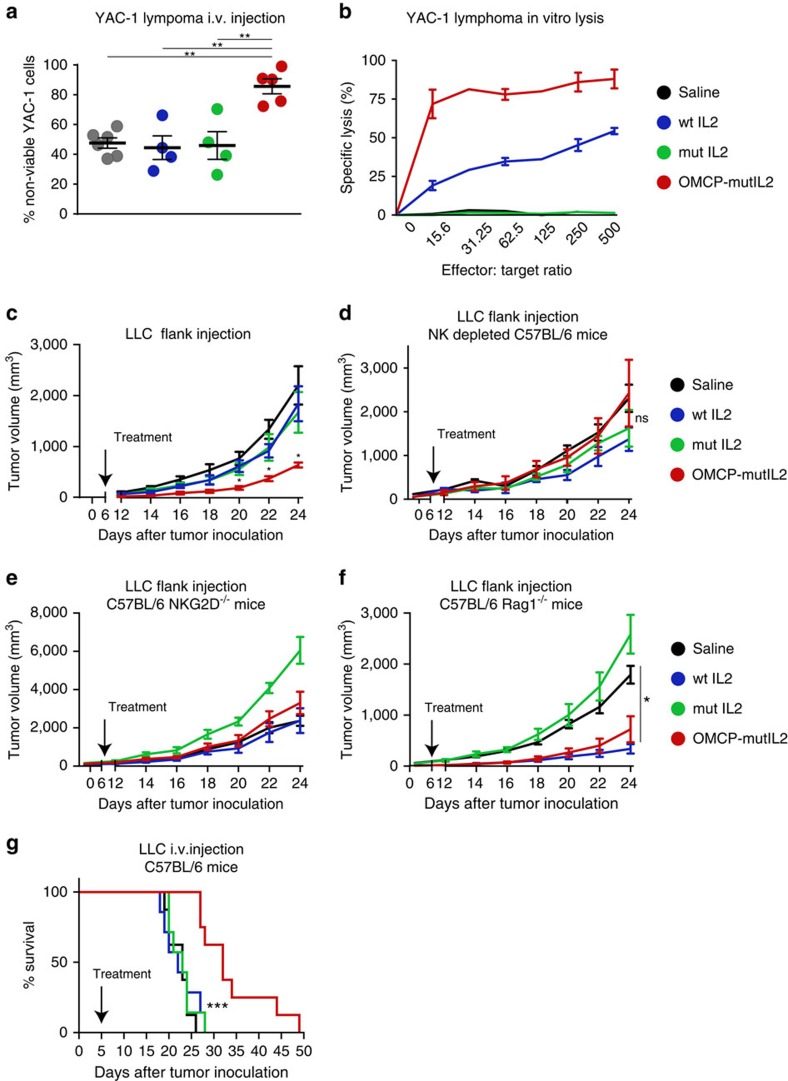
Cytokine-mediated tumour immunotherapy. (**a**) *In vivo* cytotoxicity for YAC-1 lymphoblast cell line after intravenous injection of tumour into A/J mice treated with 200,000 IUe of cytokine or OMCP-mutIL-2 construct. Comparison performed by unpaired *t*-test between groups as indicated by the lines above the graphs. (**b**) *In vitro* lysis of YAC-1 lymphoma by bulk A/J splenocytes after 200,000 IUe of cytokine or OMCP-mutIL-2 construct treatment. (**c**) LLC flank growth after cytokine treatment of wild-type C57BL/6 mice. Treatment with 750,000 IUe of cytokine or fusion construct was started on day 5-post tumour injection, after palpable tumors were evident, and continued through day 10 in 10 equal doses of 75,000 IUe/dose. Raw data are shown in Supplementary Data 1 and represents 6 mice in the saline group and 5 in the wtIL-2, mutIL-2 and OMCP-mutIL-2 group. (**d**) LLC tumour growth in C57BL/6 mice depleted of NK1.1 cells or (**e**) mutant mice deficient in NKG2D. Data representative of 5 mice per group for both NK1.1 depleted and NKG2D deficient mice. (**f**) LLC tumour growth in C57BL/6^Rag1−/−^ mice. Data representative of 5 mice per group. Comparison performed by multiple unpaired *t*-tests at each individual time point to saline-treated controls for all tumour growth experiments. (**g**) Survival of C57BL/6 mice treated with 750,000 IUe of cytokine 5 days after injection of 1 × 10^5^ LLC intravenously. Data extrapolated from groups consisting of 7 mice in the wtIL-2 and mutIL-2 treated groups and 8 mice from the saline and OMCP-mutIL-2 treated groups. Cytokines or OMCP-mutIL-2 fusion protein was given as ten doses on days 5–10 post tumor injection with A/J mice receiving a total of 200,000 IUe and C57BL/6 mice receiving 750,000 IUe. Kaplan–Meier survival graphs were compared by Log-rank (Mantel–Cox) test. ns *P*>0.05; *P<0.05; **P<0.01; ***P<0.001; black=saline; blue=wtIL-2, red=OMCP-mutIL-2, green=mutIL-2.

NK cells form the primary barrier for expansion of some malignancies, such as lymphoma and lung cancer^16^,^32^,^33^,^34^. To evaluate lymphoma clearance we intravenously injected cytokine or fusion protein-treated A/J mice with the A/J-derived CFSE-labelled YAC-1 lymphoblast cell line and evaluated their lungs 6 h later. Near complete clearance of YAC-1 was evident in OMCP-mutIL-2 treated mice while a significant number of viable lymphoblasts remained in wtIL-2, mutIL-2 and saline-treated mice (Fig. 8a and Supplementary Data 1). Similarly bulk splenocytes of A/J mice treated with OMCP-mutIL-2 lysed YAC-1 more efficiently than those treated with wtIL-2, mutIL-2 or saline (Fig. 8b). Similar results were obtained with LM-2 lung cancer targets (Supplementary Fig. 6a).

To evaluate cytokine therapy in controlling lung cancer growth we injected C57BL/6 mice in the flank with the highly aggressive Lewis Lung Carcinoma (LLC) and five days later, when palpable tumours were evident, treated them with either OMCP-mutIL-2, wtIL-2, mutIL-2 or saline. Significantly decreased tumour growth was evident after treatment with OMCP-mutIL-2 (Fig. 8c). Splenocytes from OMCP-mutIL-2 treated mice also demonstrated higher LLC cytotoxicity *in vitro* (Supplementary Fig. 6b). Increased efficacy of OMCP-mutIL-2 immunotherapy disappears in NKG2D^−/−^ or NK1.1 depleted mice (Fig. 8d,e). In fact in NKG2D^−/−^ mice mutIL-2 increased the rate of LLC growth. To further define the role of the adaptive immune system in cytokine-mediated immunotherapy we next injected LLC into C57BL/6^Rag−/−^ mice, deficient in T, B and NKT cells. In this mutant strain both wtIL-2 and OMCP-mutIL-2 were able to mitigate tumour growth over mutIL-2 or saline-treated mice (Fig. 8f). This data supports the model that OMCP-mutIL-2 mediated control of LLC growth can occur independent of T or NKT cells and confirms our *in vitro* data (Fig. 7c–f) that ‘competition' for wtIL-2 from other lymphocytes limits NK cell activation by wtIL-2.

Since intravenous injection of LLC results in pulmonary metastases and rapid animal demise we next injected C57BL/6 mice with LLC i.v. and treated them with OMCP-mutIL-2, wtIL-2, mutIL-2 or saline five days after tumour injection. Significant prolongation of survival was evident in mice treated with OMCP-mutIL-2 compared with all other conditions (Fig. 8g). Collectively, our data suggest that OMCP-mediated targeting of mutIL-2 to NKG2D expressing cells offers a safe and efficacious form of immunotherapy for both solid and liquid tumours in various strains of mice.

## Discussion

Here we describe a unique approach for IL-2 immunotherapy using a fusion protein targeting a mutant form of IL-2 to NKG2D-expressing cells. We demonstrate that this approach results in superior NK cell-mediated tumour immunotherapy with no adverse side effects associated with wild-type IL-2. Several alternative strategies have been proposed to minimize the untoward effects of IL-2 and preferentially activate cytotoxic lymphocytes. One strategy has been to create mutants with increased affinity for IL-2Rβ to remove the preference for IL-2Rα^35^,^36^. Our results suggest that competition with IL-2Rα-expressing cells limits bioavailability of wtIL-2 to cytotoxic lymphocytes. The IL-2Rβ-enhanced IL-2 mutants would still bind and activate T_regs_ and vascular endothelium, potentially limiting their efficacy due to ‘off target' IL-2 signalling.

Another potential way to overcome side effects involves administration of anti-IL-2 antibodies that sterically inhibit wtIL-2 binding to IL-2Rα^1^,^37^,^38^. Such treatment can extend serum half-life^29^ due to the Fc region of the antibody and potentially due to reduced competition for wtIL-2 from IL-2Rα-expressing cells. However, IL-2Rα blockade could be incomplete due to dissociation of antibody from IL-2. Additionally antibody-IL-2 fusion proteins also have been designed to target IL-2 to specific tumour antigens^39^,^40^. This approach could be limited by tumour-mediated alteration of the targeted antigen under selective pressure of the antibody-IL-2 fusion protein^41^.

IL-2 mutants with reduced affinity for IL-2Rα have also been tested. Compared with wtIL-2 these mutants can be administered in supratherapeutic doses without IL-2Rα-mediated capillary leak or systemic toxicity^42^. While these mutants have excellent safety profiles, they activate cytotoxic lymphocytes poorly (Fig. 4b,d)^43^. Our approach combines these concepts to target a safe form of IL-2 directly to cytotoxic lymphocytes, rather than tumours. This is accomplished by replacing the normal targeting of IL-2 to IL-2Rα with NKG2D. The combination of an IL-2Rα-deficient IL-2 fused to a high affinity NKG2D-ligand improves upon previous strategies by specifically expanding NK cells without any apparent activation of T_regs_ or accumulation of extracellular fluid. These findings offer the promise of a potentially safe and highly efficacious form of IL-2.

One limitation in translating results from inbred lab animals to humans is the natural diversity in cytokine reactivity and threshold for lymphocyte activation. Previous studies have demonstrated a correlation between *ex vivo* lymphocyte cytotoxicity and cancer immunity^44^. Therefore, any potential therapy needs to account for a population that has differential levels of cytotoxic lymphocyte activity and reactivity. For example, NK cells from C57BL/6 mice are activated by wtIL-2 and high doses of mutIL-2. In contrast, wtIL-2/anti-IL-2 antibody complexes result in expansion of NK cells in A/J but not C57BL/6 mice (Fig. 4b versus d). Such variations highlight the limitations of translating results derived from a single strain of mice to immunologically diverse humans. Of note, OMCP-mutIL-2 expanded NK cells in both strains of mice indicating that this therapy could be efficacious in populations with diverse NK function and reactivity.

As OMCP has been described as a competitive antagonist of NKG2D activation its use may be construed as counterproductive for tumour immunotherapy^14^,^15^. Nevertheless, natural cytotoxicity and tumour clearance were augmented in OMCP-mutIL-2-treated mice even in the presence of established tumours. NKG2D signal transduction *in vitro* was relatively preserved as well (Supplementary Fig. 4e). This suggests minimal or transient NKG2D receptor occupancy and preservation of its signalling capacity. Alternatively, shed NKG2D ligands may promote tumour immunity through reversal of NK desensitization imposed by chronic agonist engagement^45^. It is thus possible that within the tumour bed such competitive antagonism plays a paradoxical role in NK activation. OMCP thus presents an ideal ‘targeting vector' due to its high affinity and long half-life of binding to NKG2D.

Immunogenicity can be an obstacle to successful therapy with cytokine mutants. Anti-protein antibodies can neutralize the cytokine effect on repeat administration and T cell-specific antigens may induce cellular immunity as well. For this reason we have chosen a virally encoded delivery system which has co-existed and co-evolved with man for immunoevasion^14^. While previous clinical trials of IL-2 mutants did result in the detection of anti-mutant IL-2 antibodies in some patients, no adverse side effects were associated with this immune response^46^. Nevertheless future work may need to be done to identify and modify any immunogenic determinants in OMCP-mutIL-2 prior to translation to clinical applications in man.

While NK cells from two separate strains of mice were activated by OMCP-mutIL-2 we did not detect global expansion or activation of T cells *in vivo*, although CD8^+^ T cells were activated by higher concentrations in *vitro* (Fig. 2c) or after the addition of a T cell receptor agonist (Supplementary Fig. 2c). NKT cells were activated *in vivo* by OMCP-mutIL-2, albeit to a lesser extent then NK cells (Supplementary Fig. 4b,d). Such effects could be the result of differences in thresholds for cytokine activation of different lymphocyte subsets in the absence of additional activating receptor stimulation. Alternatively it is possible that NK cell are constitutively activated through endogenously present stress ligands on dead or dying cells while T cells receive little receptor stimulation in the absence of exogenously administered agonists. Based on our phenotypic findings of selective NK expansion we focused on immunotherapy for lung cancer and lymphoma, whose growth is restricted primarily by NK cells^16^,^33^,^34^,^47^. However NKG2D-targeted delivery of immunostimulatory cytokines may lead to the expansion and/or activation of both NK and antigen-specific CD8^+^ T cells and NKT cells in the presence of appropriate T cell receptor stimulation. Future work will focus on tumour models containing tumour-associated antigens in order to study the interplay of the innate and adaptive immune system after targeted delivery of IL-2.

## Methods

### Cytokine and construct generation

The sequences encoding human IL-2 (1–133; C125S) and mutant IL2 (1–133; R38A, F42K, C125S) (GTGSSGSSDYKDDDDKHHHHHHHHGSSGSSAPTSSSTKK TQLQLEHLLLDLQMILNGINNYKNPKLAMLTKKFYMPKKATELKHLQCLEEELKPLEEVLNLAQSKNFHLRPRDLISNINVIVLELKGSETTFMCEYADETATIVEFLNRWITFSQSIISTLT) were cloned into the pFM1.2R(ref. 41) with an N-terminal FLAG/hexahistidine tag. The chimeric OMCP-mutIL-2 molecule comprises the full-length OMCP (1–152) coding sequence cloned in frame with mutant IL-2 (1–133; R38A, F42k, C125S) joined by a glycine/serine linker (OMCP-mIL2: HKLAFNFNLEINGSDTHSTVDVYLDDSQIITFDGKDIRPTIPFMIGDEIFLPFYKNVFSEFFSLFRRVPTSTPYEDLTYFYECDYTDNKSTFDQFYLYNGEEYTVKTQEATNKNMWLTTSEFRLKKWFDGEDCIMHLRSLVRKMEDSKRNTGGTGSSGSSDYKDDDDKHHHHHHHHGSSGSSAPTSSSTKKTQLQLEHLLLDLQMILNGINNYKNPKLTAMLTKKFYMPKKATELKHLQCLEEELKPLEEVLNLAQSKNFHLRPRDLISNINVIVLELKGSETTFMCEYADETATIVEFLNRWITFSQSIISTLT) cloned into the pFM1.2R vector. Proteins were expressed by transient transfection into FreeStyle 293-F cells (Life Technologies). Supernatant was recovered at 72 and 144 h post-transfection. Supernatants were supplemented with 5 mM imidazole and 0.02% sodium azide and purified by nickel-nitrilotriacetic acid (Ni-NTA) chromatography (Gold-Bio). Purified proteins were buffer exchanged into phosphate-buffered saline and flash frozen in liquid nitrogen. Equivalent *in vitro* and *in vivo* activity was documented for wild-type IL-2 generated in house and Teceleukin (Tecin™) available from the NCI repository (Frederick National Laboratory for Cancer Research). Thus, for some experiments these two preparations of IL-2 were used interchangeably.

Wild-type IL-2 has a specific activity of 15 × 10^6^ IU mg^−1^ (ref. 48). Thus, based on the molecular weight of 15.5 kDa a 4.4 μM solution is equivalent to 1,000 IU μl^−1^. Based on this calculation all cytokines and construct were administered on a molar basis with 1 μl of 4.4 μM solution defined as 1,000 IU equivalents (IUe from here on). Such a system allows for equimolar comparison between IL-2, mutIL-2 and OMCP-mutIL-2 despite difference in molecular weight.

### Animals

Male A/J, C57BL/6J, C57BL/6^CD45.1+^ and C57BL/6^*Rag1*−/−^ mice 8–12 weeks of age were purchased from The Jackson Laboratory (Bar Harbour, Maine). NKG2D^−/−^ mice on the B6 background were kindly provided by Wayne Yokoyama (Howard Hughes Institute of Medicine at Washington University in St Louis) and bred in house. Animals were housed in a barrier facility in air-filtered cages and allowed free access to food and water. For some experiments A/J mice were treated with depleting concentrations of anti-Asialo-GM1 (50 μl day −2; 25 μl day −1) or control rabbit IgG (Wako Chemical Company). Animal procedures were approved by the animal studies committee of Washington University School of Medicine, St Louis, MO.

### Tissue harvest and *in vitro* cultures

Single cell suspensions of splenocytes were obtained by crushing whole spleens through 70 μm cell strainers prior to RBC lysis by ACK buffer (Lonza, Walkersville, MD) and re-filtration through a 40 μm filter. Lungs were digested for 90 min at 37 °C in 1 mg ml^−1^ collagenase II (Fisher Scientific), and 5 U ml^−1^ DNase I (Sigma-Aldridge) prior to processing in an identical fashion to spleens.

For *in vitro* cultures splenocytes from either A/J, B6, or NKG2D^−/−^ mice were extracted in a sterile fashion and seeded in 12-well plates in complete media (RPMI 1640 supplemented with 10% FBS, 100 U ml^−1^ Penicillin and Streptomycin, 2 mM L-glutamine and 50 μM 2-Mercaptoethanol) at 5 million cells per ml per well. The cells were treated with increasing doses of human recombinant IL-2, mutIL-2, OMCP-mutIL-2, or OMCP for 36 h as described in the manuscript. For some experiments bulk splenocytes were labelled with CFSE and cultured in 1000 IUe ml^−1^ of cytokine for 5 days prior to flow cytometric analysis. For NK isolation experiments bulk splenocytes were processed using either the NK cell isolation kit II or CD49b (DX5) positive magnetic bead selection (both from Miltenyi Biotech). For STAT5 phosphorylation experiments isolated NK cells were stimulated in increasing concentrations of IL-2 or construct at 100,000 cells per 500 μl for 15 min. For experiments evaluating the interaction of NK cells with splenic stroma DX5 positively selected NK cells were labelled with CFSE (for identification after fixation and permeabilization) and recombined with NK-depleted stromal cells at a 5%/95% NK/stromal cell ratio. As described in the manuscript for some studies NKG2D^−/−^ NK cells were combined with wild-type splenic stromal cells. For other experiments NK-depleted splenocytes from wild-type B6 mice were treated with saturating concentrations of anti-IL-2α blocking antibody (clone 3C7) or isotype control (both from Biolegend) prior to recombining with NK cells. For such competitive STAT5 phosphorylation experiments 100,000 cells (including 5% CFSE-labelled NK cells) were resuspended into 2 μl complete media containing 1,000 IUml^−1^ of either wtIL2, mutIL-2 or OMCP-mut-IL-2 (freshly prepared and pre-warmed). The cells were then incubated at 37 °C for 15 min.

Human PBMC were collected under IRB approved protocol at Washington University in St Louis. Informed consent was obtained from all volunteers donating blood. For non-antigen specific cytokine driven expansion and activation, freshly isolated PBMC were co-cultured for 72 h with appropriate concentration of cytokines and lymphocyte phenotype determined flow cytometrically as described in each figure. For antigen-specific stimulation, PBMC from CMV-seropositive donors were cultured with pp65 peptide and the indicated concentration of cytokine. Two weeks after culture initiation, cells were harvested and stained with anti-CD8 Ab and HLA-A*0201/CMV pp65 peptide tetramers and analysed by flow cytometry.

### Flow cytometry

All flow cytometric analysis was performed using saturating concentrations of fluorochrome-conjugated antibodies at 4 °C in FACS buffer consisting of PBS with 2% FBS and 0.4% EDTA. All antibodies were anti-mouse and purchased from BD Bioscience or eBioscience. Unless otherwise indicated in the antibody data sheet all staining was performed by adding 1 μl of the fluorochrome-conjugated antibody to 1–2 × 10^6^ splenocytes and stained at 4 °C for 30–45 min in 100 μl FACS buffer consisting of phosphate buffered saline with 5% fetal calf serum. Excess antibody was removed by two consecutive washings. Antibodies used for this study consisted of anti-CD4 (clones GK1.5 or RM4–5), anti-CD8 (clone 53-6.7), anti-CD278 (ICOS) (clone: 7E.17G9), anti-CD25 (clone PC61), anti-KLRG1 (clone 2F1), CD49b (Integrin alpha 2) (clone DX5), anti-CD3e (clone 145-2C11), anti-CD45 (clone 30-F11), anti-CD69 PE (clone H1.2F3), anti-GITR (clone DTA-1), anti-Foxp3 (clone: FJK-16s) and Anti-Stat5 (clone 47/Stat5; pY694). Antibodies were conjugated to either FITC, PE, PerCP-Cy5.5, PE-Cyanine7, APC, APC-eFluor 780, eFluor 450 or Alexa Fluor 647. Human peripheral blood leucocyte analysis was carried out similar to murine splenocyte data with some modifications. Specifically perforin staining was carried out using the purified mouse anti-human perforin (clone δG9) from BD Bioscience. After surface staining the leucocytes were fixed and permeabilized with BD fixation and permeabilization solution. The cells were stained with fluorochrome-conjugated anti- perforin or isotype control per manufacturer instructions. Additional human antibodies consisted of mouse anti-human CD56 (clone MY31), anti-CD3 (clone SK7), anti-CD8 (SK1) and anti-CD4 (SK3) from BD Bioscience.

Phospho-STAT5 evaluation was performed by paraformaldehyde fixation, methanol permeabilization, followed by staining with AlexaFluor488-conjugated Anti-Stat5 (pY694) (BD Pharmingen; clone 612599). To accomplish this isolated NK cells were combined with NK-depleted splenic stromal cells and fixed in 2% paraformaldehyde at 37 °C for 10 min after IL-2 stimulation for 15 min at 37 °C. The cells were then washed once with ice-cold PBS and permeabilized by adding 0.5 ml per tube of 90% methanol on ice for 1 h. Methanol was removed and the cells were washed once with ice-cold PBS (to wash away the excess methanol), and stained for 1 h with anti-Stat5 (pY694) antibody at room temperature followed by one wash in PBS containing 0.5% fetal calf serum and 0.4% EDTA.

For analysis of construct and cytokine binding to lymphocytes recombinant wtIL-2, mutIL-2 and OMCP-mutIL-2 were biotin labelled using EZ-Link NHS-PEG_4_-Biotin (Thermo Fischer Scientific) following manufacturer's instruction. Mouse spleen cells were first incubated with FACS buffer or unlabelled OMCP at 0.8 μM followed by staining with each of the biotin-labelled IL-2 proteins at 0.15 μM and a mixture of fluorescence conjugated antibodies in Fc block in two steps. Finally the cells were stained with PE-streptavidin. All staining was carried out on ice with 30 min incubation for each step. Stained samples were analysed by flow cytometry using BD LSRFORTESSA X-20 and the data were analysed with FlowJo software (Tree Star). NK cells were defined as viable, NK1.1^+^CD3^−^CD19^−^.

### Western blot analysis of signalling pathways

Murine NK cells were purified from C57BL/6 male spleens immediately prior to the experiment according to the procedure described elsewhere in this manuscript. KY1 (murine) cell line was maintained in RPMI-1640 medium without serum and IL2 for 4 h prior to the experiment. Hamster anti-mouse NKG2D/CD314 clone A10 (manufactured in house) were immobilized in PBS on 6-well tissue culture plates (TPP, Midwest Scientific, Valley Park, MO) either overnight at 4 °C or for 3 h at 37 °C. The wells were washed once with PBS followed by the addition of 1000 IUe ml^−1^ of OMCP-mutIL-2, wtIL-2, and mutIL-2 and 2 mM sodium pervanadate (Sigma, St Louis, MO) to the appropriate wells, except the NKG2D well. Next, 5 × 10^6^ KY1.1 cells or 1 × 10^6^ murine NK cells were added to the wells and plates incubated at 37 °C for 5 min. The cells were lysed *in situ* for 30 min at 4 °C by the addition of 10 × cell lysis buffer containing 20 mM Tris-HCl (pH 7.5), 150 mM NaCl 1 mM Na_2_EDTA, 1 mM EGTA, 1% Triton X-100, and protease inhibitors cocktail. The resulting cell lysates were cleared by centrifugation at 14,000 r.p.m. For JAK1 phosphorylation experiments lysates were immunoprecipitated overnight with anti-JAK1 clone A-9 monoclonal antibody (Santa Cruz Biotechnology, Dallas, TX). Bulk lysates from freshly isolated NK cells were evaluated for phospho-Vav by the anti-p-Vav (Tyr 174) rabbit polyclonal antibody (Santa Cruz Biotechnology, Dallas, TX) without co-immunoprecipitation. The immune complexes were adsorbed concomitantly with 20 μl Protein G Plus agarose (Santa Cruz Biotechnology, Dallas, TX). After three washes with cell lysis buffer, the proteins were resolved on a 10% Tris-Glycine SDS–polyacrylamide gel electrophoresis (SDS-PAGE) gel (Bio-Rad, Hercules, CA) and transferred to an Immobilon-P polyvinylidene fluoride (PVDF) membrane. Next, the membranes were blocked with 3% BSA in PBS with 0.1% Tween-20 for 1 h at 20 °C. Phoshoproteins were then detected with either rabbit anti-pVav or goat anti-pJAK1 antibodies (both from Santa Cruz Biotechnology, Dallas, TX) followed by the appropriate secondary antibodies conjugated to horseradish peroxidase. The horseradish peroxidase activity was detected with Pierce enhanced chemiluminescence substrate (ThermoFisher Scientific, Waltham, MA) according to the manufacturer's instructions. The chemiluminescence signal was acquired with the ChemiDoc-MP Immaging System and analysed with the Image Lab 5.1 software application (both from Bio-Rad, Hercules, CA). Images presented throughout the manuscript have been cropped for presentation. Full size images are presented in Supplementary Fig. 7.

### LacZ assay

The BWZ.36 thymoma cell line, in which the expression of a transfected heterologous Escherishia coli B-galactosidase (lacZ) gene is controlled by the nuclear factor in activated T cells (NFAT), was generously provided by Dr Nilabh Shastri (University of California, Berkeley). The mouse NKG2D short isoform and DAP12 (wildtype or Y2F mutant) were transduced into BWZ.36 cells by retroviral vector pMX.IRES.Bs and pMIG, respectively as reporter cells for NKG2D signalling. 4–5 × 10^4^ per well of these cells in 100 μl complete RPMI1640 were cultured in the presence of 1000 U ml^−1^ wtIL2, mutIL2 or OMCP-mutIL2 for 16 h at 37 °C before lysed by Z buffer (9 mM MgCl_2_, 0.125% NP40 in PBS) containing 150 μM chlorophenol red-β-D-galactopyranoside, a substrate of lacZ. After a 6 h incubation at 37 °C absorbance of each well was measured by 96-well plate reader at OD595 nm. Cells cultured with PMA (10 ng ml^−1^) and ionomycin (0.2 μg ml^−1^) served as a positive control for NFAT-lacZ activation in each cell line, while cells cultured in the wells pre-coated with anti-NKG2D antibodies (A10, at 12 μg ml^−1^) served as a control for NKG2D signalling.

### *In vitro* cytotoxicity

^51^Chromium release was conducted by incubating the target cells with 100 μCi sodium ^51^chromate (PerkinElmer) for 1 h. Bulk splenocytes were used as effector cells and incubated with targets at defined effector:target ratios for 4 h at 37 °C in round bottom 96 well plates. Specific lysis was expressed as (experimental release-spontaneous release)/(maximum release-spontaneous release) × 100% with 0% specific lysis as lowest expressed value.

### *In vitro* T_reg_ suppression

CD90.2^+^ T lymphocytes from ‘responder' CD45.1^+^ C57BL/6 mice were isolated from bulk splenocytes using magnetic beads (Miltenyi Biotech), labelled with CFSE, and combined with CD90.2-depleted splenocytes from C57BL/6^CD45.2+^ congenic mice at a 1:1 ratio in U bottom 96 well plates. Anti-CD3 Ab (clone 145-2C11; BD Pharmingen) at 3 μg ml^−1^ final concentration was added to the cultures. T_regs_ from CD45.2^+^ congenic mice, expressing GFP on a Foxp3 promoter, were flow-cytometrically sorted from splenocytes and lymphocytes after cytokine administration as described in the text. T_regs_ were added to the co-cultures in a 1:1, 0.5:1, 0.25:1, 0.125:1 and 0:1 ratio in 200 μl final volumes of media. Seventy hours later proliferation of responder T cells was determined flow-cytometrically based on dilution of CFSE in CD45.1^+^CD45.2^−^ T lymphocytes.

### *In vivo* cytokine injections

For *in vivo* experiments the mice received intraperitoneal injections of cytokines in 200 μl volume. Dilutions were made from a stock solution of 44 μm, which is equivalent to 10,000 IUe μl^−1^. Thus for A/J mice, which received ten equal doses of 20,000 IUe per dose for a total of 200,000 IUe over the course of five days, 2 μl were resuspended in 198 μl of saline for injection. For C57BL/6 mice, which received 10 equal doses of 75,000 IUe per dose for a total of 750,000 IUe over the course of five days, 7.5 μl were resuspended in 192.5 μl of saline for injection. All cytokines were given in 10 equal doses twice a day (BID) over a period of five days to total the cytokine described in the results. As described above all cytokines were normalized to IUe on a molar basis. For select experiments, the mice were then killed on day 6 and organs were fixed in 10% buffered formalin for histological analyses. For other experiments splenocyte and lung lymphocyte populations were analysed flow cytometrically. For all the *in vivo* cytokine treatment experiments, animals were weighted (daily or every other day) and expressed as % change from start of cytokine therapy.

For evaluation of serum concentration wtIL-2, mutIL-2 or OMCP-mutIL-2 were labelled with Alexa Fluor 647 (LifeTechnologies Inc.) according to manufacturer instructions. Serum was collected at times specified and concentration of cytokine determined fluoroscopically according to a standard curve.

### *In vivo* tumour studies

Lewis lung carcinoma (LLC) cells were subcutaneously injected into C57BL/6, C57BL/6^NKG2D−/−^ or C57BL/6^*Rag1*−/−^ mice at 1 × 10^5^ cells per mouse in 100 μl of sterile saline. Once visible tumours were evident, day 5 post-injection, a five-day course of cytokine treatment (given in BID doses) was started as described above. Measurement of cross sectional tumour diameter was performed using calipers and tumour volume estimated as 4/3πr^3^. The mice were killed on day 24 post injection. Animals were monitored every 2 days, starting 6 days after injection and tumour size recorded. Animals were killed if tumours measured >20 mm in any direction, or if they showed signs of distress or loss of >15% of their body weight. Supplementary Data 1 shows the raw data for tumour measurements shown in Fig. 8c. One tumour in the IL-2, mutIL-2 and OMCP-mutIL-2 from experiment shown in Fig. 8c, developed a skin ulcer within three weeks of injection and was excluded from final analysis. Permission was obtained from the veterinarian, on an individual basis, to maintain animals with skin ulcers over tumours. Once any animal in any group was recorded as having a tumour measuring >20 mm in diameter, all animals were killed and the study terminated. For NK cell depletion, mice were treated with anti-NK1.1 antibody (clone PK136) or mouse IgG isotype control (both from BioXcell) at 500 μg day −2, 250 μg day −1 and 250 μg weekly for the duration of the experiment. For lymphoma clearance experiments A/J mice were treated with ten doses of cytokine over a period of 5 days as described above and on day 6 injected intravenously with YAC-1 cells that were labelled with CFSE at 5 × 10^6^ cells per /mouse. Mice were killed 4 h later, lungs were digested and viability of YAC-1 determined by forward and side scatter analysis of CFSE^+^ cells.

### Statistics

Comparison of splenic and lymphocytes between various cytokine treatment conditions was performed by unpaired *T*-test with Welch's correction to account for unequal variance or unequal sample size. Tumour growth between different cytokine conditions was compared by multiple unpaired-T tests performed between various conditions at various time points using the Sidak–Bonferroni correction. Fold change in STAT5 phosphorylation was evaluated by unpaired *T*-test with Welch's correction in a similar fashion. *In vitro* lymphocyte activation and proliferation was performed by paired *T*-test across the same experimental conditions. Comparison of multiple groups performed by analysis of variance (ANOVA) where indicated.

### Data availability

The data that support the findings of this study are contained within the Article or Supplementary Files, or available from the authors on request.

## Additional information

**How to cite this article:** Ghasemi, R. *et al*. Selective targeting of IL-2 to NKG2D bearing cells for improved immunotherapy. *Nat. Commun.* 7:12878 doi: 10.1038/ncomms12878 (2016).

## Supplementary Material

Supplementary Information Supplementary Figures 1-7

Supplementary Data 1 Treatment of LLC tumors in C57BL/6J mice with different IL2 constructs
