# Supplementary material for: Selective targeting of IL-2 to NKG2D bearing cells for improved immunotherapy
Source: Nat Commun. 2016 Sep 21;7:12878. doi: 10.1038/ncomms12878 (PMC5036003; doi:10.1038/ncomms12878)
Supplement: Supplementary Information — Supplementary Figures 1-7 [file ncomms12878-s1.pdf]

Supplementary Figure 1

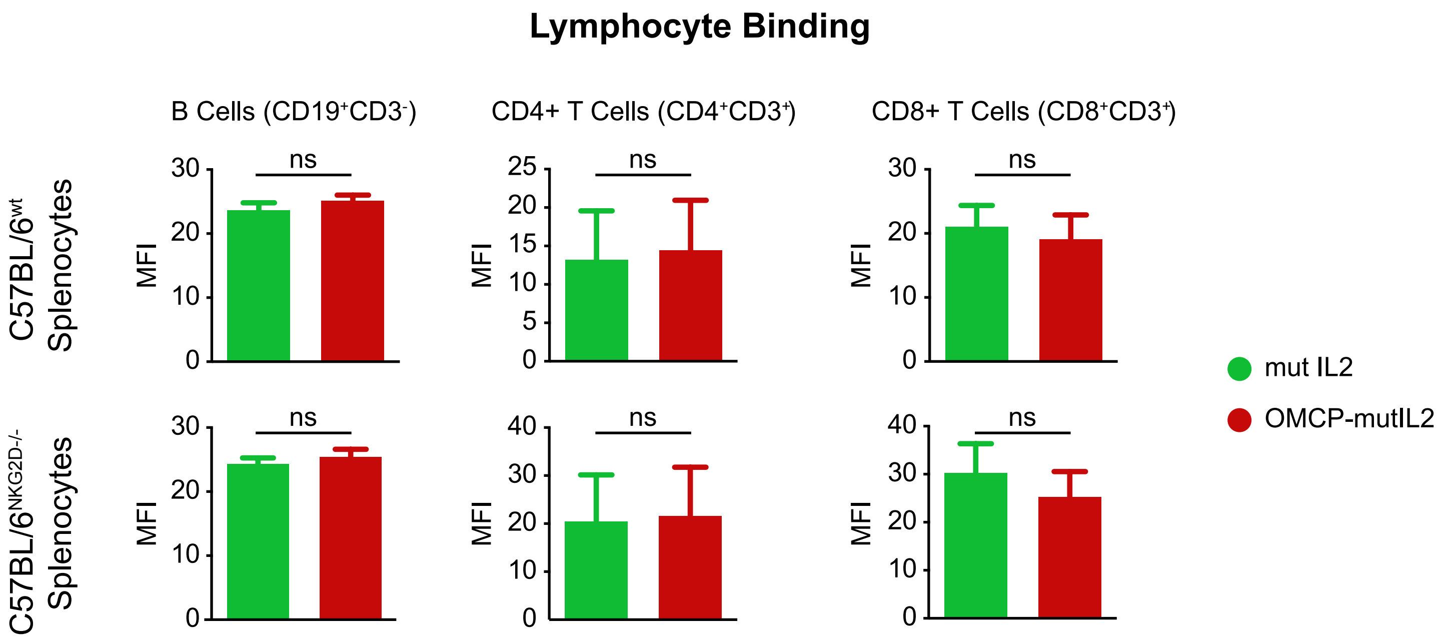

Supplementary Figure 1: Cytokine binding of various lymphocyte subsets in vitro. Binding of biotin-labeled mutIL-2 or OMCP-mutIL-2 to various splenocyte subsets in vitro.. As described in the methods section all cells were co-cultured with equimolar concentration of biotinylated cytokines and fusion construct at 4°C followed by labeling with phycoerythrin (PE) conjugated streptavidin and fluorochrome-conjugated antibodies for various cell surface receptors. Data was acquired flow cytometrically and is representative of three separate experiments with MFI comparison of PE-labeled constructs. Data comparison performed by unpaired t-test for individual comparisons and shown as mean±SEM. All bar graphs represent mean±SEM. ns p>.05; red=OMCP-mutIL-2, green=mutIL-2.

Supplementary Figure 2

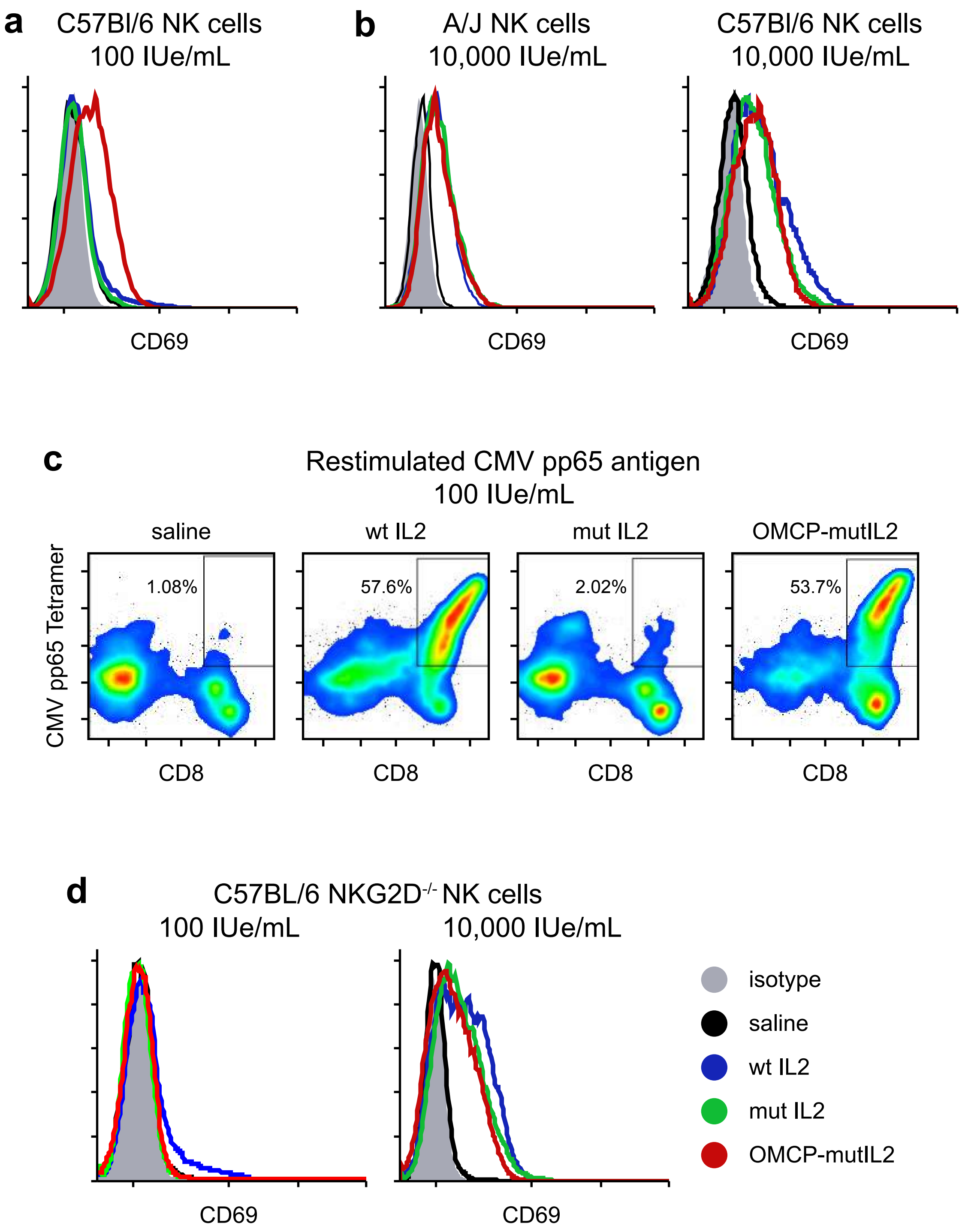

Supplementary Figure 2: **Activation of A/J and C57BL/6 NK cells**. *In vitro* activation of C57BL/6 or A/J NK cells after 36 hours of culture in (**a**) 100 IUe or (**b**) 10,000IUe/ml of cytokines or OMCP-mutIL-2 construct. (**c**) Expansion of CMV pp65-specific human $CD8^+$ T cells from PBMC upon peptide / IL-2 stimulation. Numbers represent frequency of pp65-specific T cells in $CD8^+$ gated cells. Data is representative of two independent experiments performed from separate donors (**d**). *In vitro* activation of $NKG2D^{-/-}$ C57BL/6 NK cells after 36 hours of culture in 100 or 10,000 IUe/ml of cytokines or OMCP-mutIL-2 construct. Representative of 2 to 3 experiments per condition.

Supplementary Figure 3

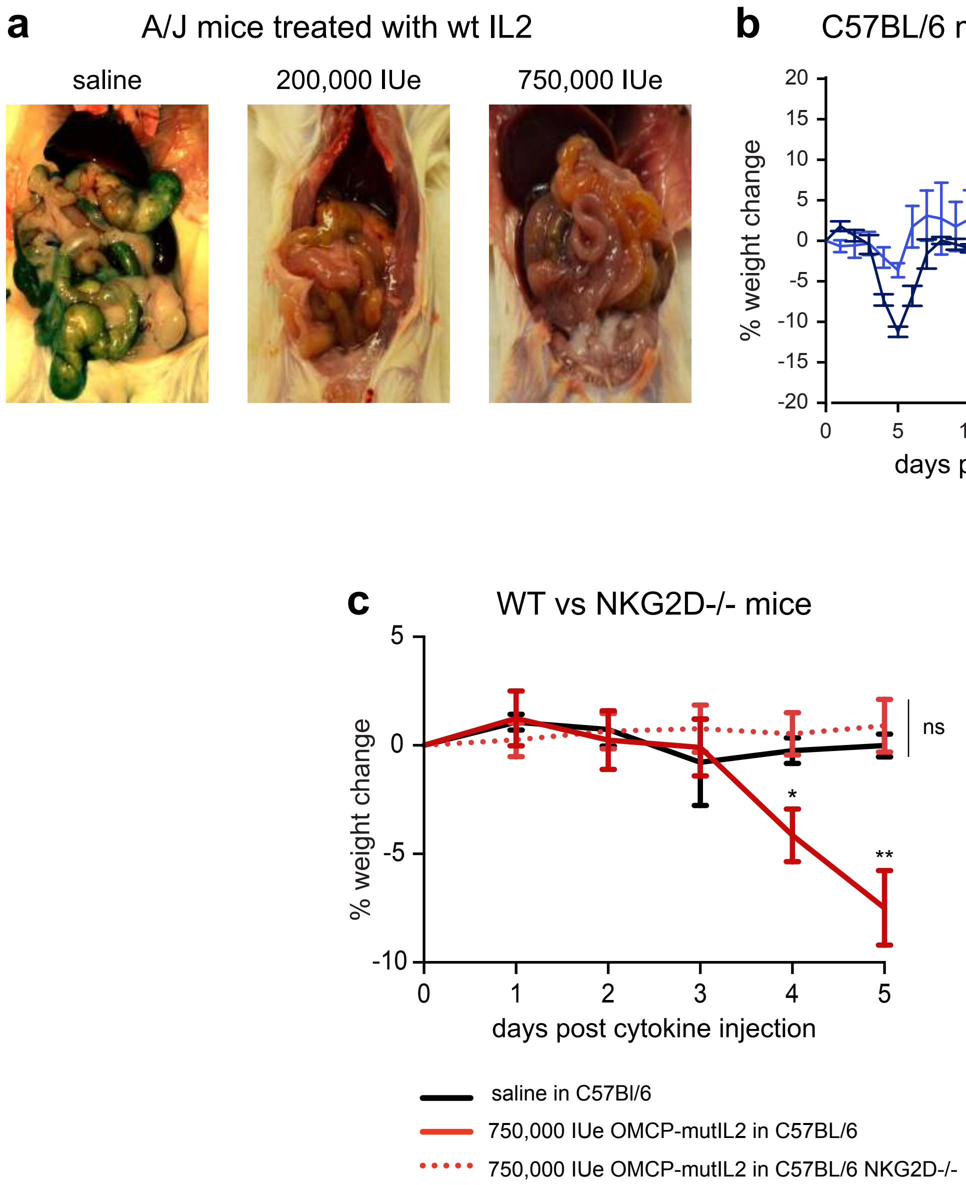

Supplementary Figure 3: Weight loss and anorexia in wtIL-2-treated mice. (**a**) Inspection of the viscera demonstrates limited food consumption after a 5-day course of 200,000 or 750,000 IUe of wtIL-2 in A/J mice. (**b**) Unlike the A/J strain B6 mice are able to tolerate higher doses of wtIL-2 with only moderate weight loss after 750,000 IUe. Higher doses of 1,500,000 IUe wtIL-2 resulted in increased weight loss. Doses above this regimen led to animal death. Representative of three separate experiments representing 5-7 mice per group. **(c)** Weight loss in C57BL6 wild-type mice treated with 750,000 IUe of OMCP-mutIL2 (solid red line), C57BL6 wild-type mice treated with saline (black line) or C57BL6$^{NKG2D-/-}$ mice treated with 750,000 IUe of OMCP-mutIL2 (dotted red line). Weight loss compared by unpaired t-test at each individual day. ns $p>.05$; * $p<.05$

Supplementary Figure 4

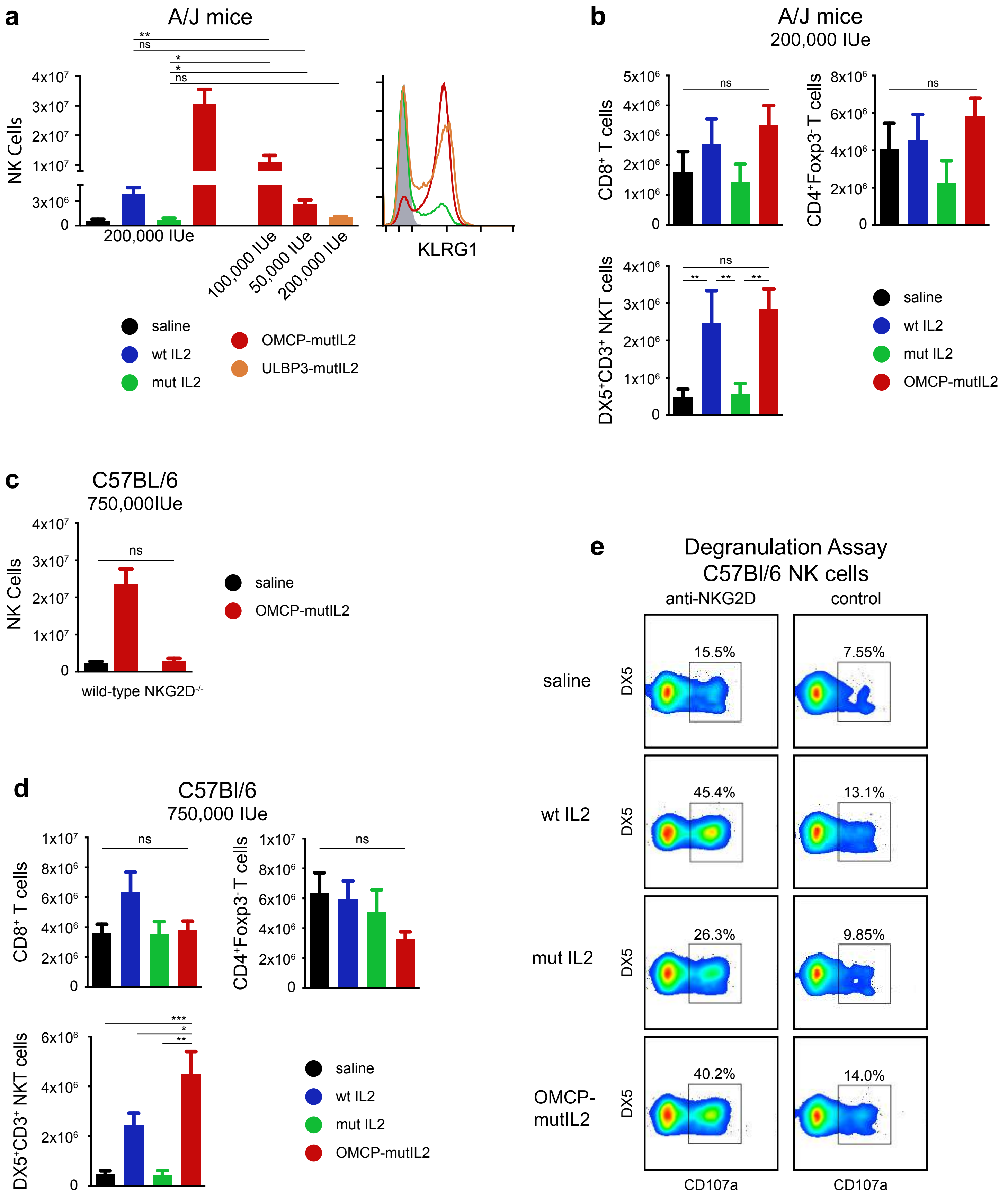

Supplementary Figure 4: Alternative methods of IL-2 delivery (**a**) NK expansion by lower doses of OMCP-mutIL-2 or ULBP3-mutIL-2 in A/J spleen (left panel). NK activation, as measured by surface KLRG1 expression on NK cells treated with 200,000 IUe of mutIL-2 (green), OMCP-mutIL2 (red), or ULBP3-mutIL-2 (orange) in A/J spleen (right panel). All graphs represent an average cell count ± SEM from 5-6 mice per group performed as 4-5 separate experiments for each strain of mice. Comparison performed by unpaired t-test between groups as indicated by the lines. (**b**) Expansion of $CD8^+$, $CD4^+$ $Foxp3^-$ T cells or $DX5^+CD3^+$ NKT cells in the spleens of A/J mice treated with 200,000 IUe of mutIL-2 (green), OMCP-mutIL2 (red) or wtIL-2 (blue). (c) Expansion of splenic NK cells in OMCP-mutIL-2 treated C57BL/6 wild-type vs. C57BL/$6^{NKG2D-/-}$ mice. (**d**) Expansion of $CD8^+$, $CD4^+$ $Foxp3^-$ T cells or $DX5^+CD3^+$ NKT cells in the spleens of C57BL/6 mice treated with 750,000 IUe of mutIL-2 (green), OMCP-mutIL2 (red) or wtIL-2 (blue). (**e**) Degranulation of C57Bl/6 NK cells to plate bound NKG2D as measured by surface CD107a expression in the presence of saline, mut-IL-2, wtIL-2 or OMCP-mutIL-2 at 1000 IUe/ml.

Supplementary Figure 5

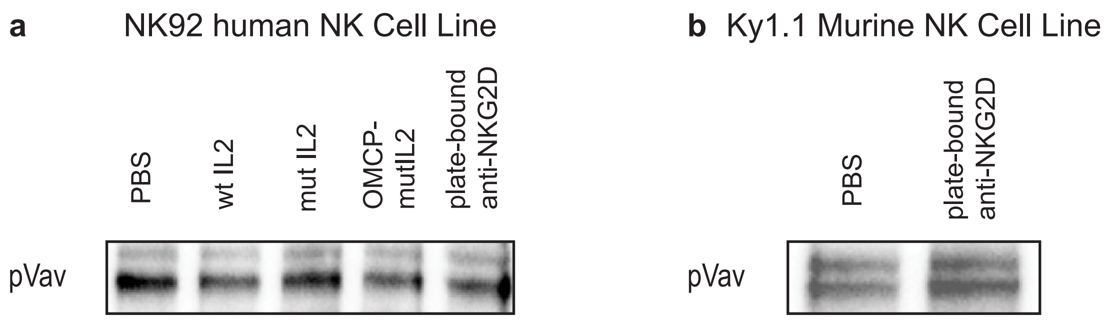

Supplementary Figure 5: Vav Phosphorylation in NK cells lines. Both NK92 human (a) and Ky1.1 murine (b) NK cell lines demonstrate constitutive Vav phosphoryation as determined by Western blot comparison of phosphate-buffered saline (PBS) vs. plate-bound NKG2D.

Supplementary Figure 6

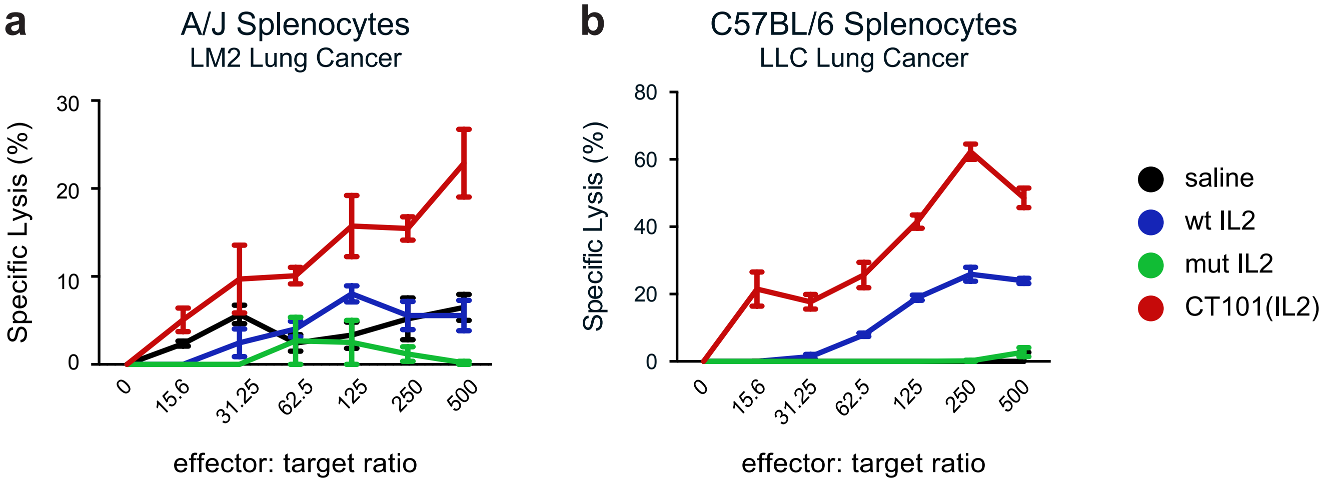

Supplementary Figure 6: *In vitro* lysis of tumor cell lines (**a**) *In vitro* lysis of A/J-derived LM2 lung adenocarcinoma by bulk splenocytes after a five day course of 200,000 IUe of cytokine given over ten doses. (**b**) *In vitro* lysis of LLC lung cancer by B6 splenocytes treated with 750,000 IUe of cytokines or constructs given over five days in ten doses.

Supplementary Figure 7

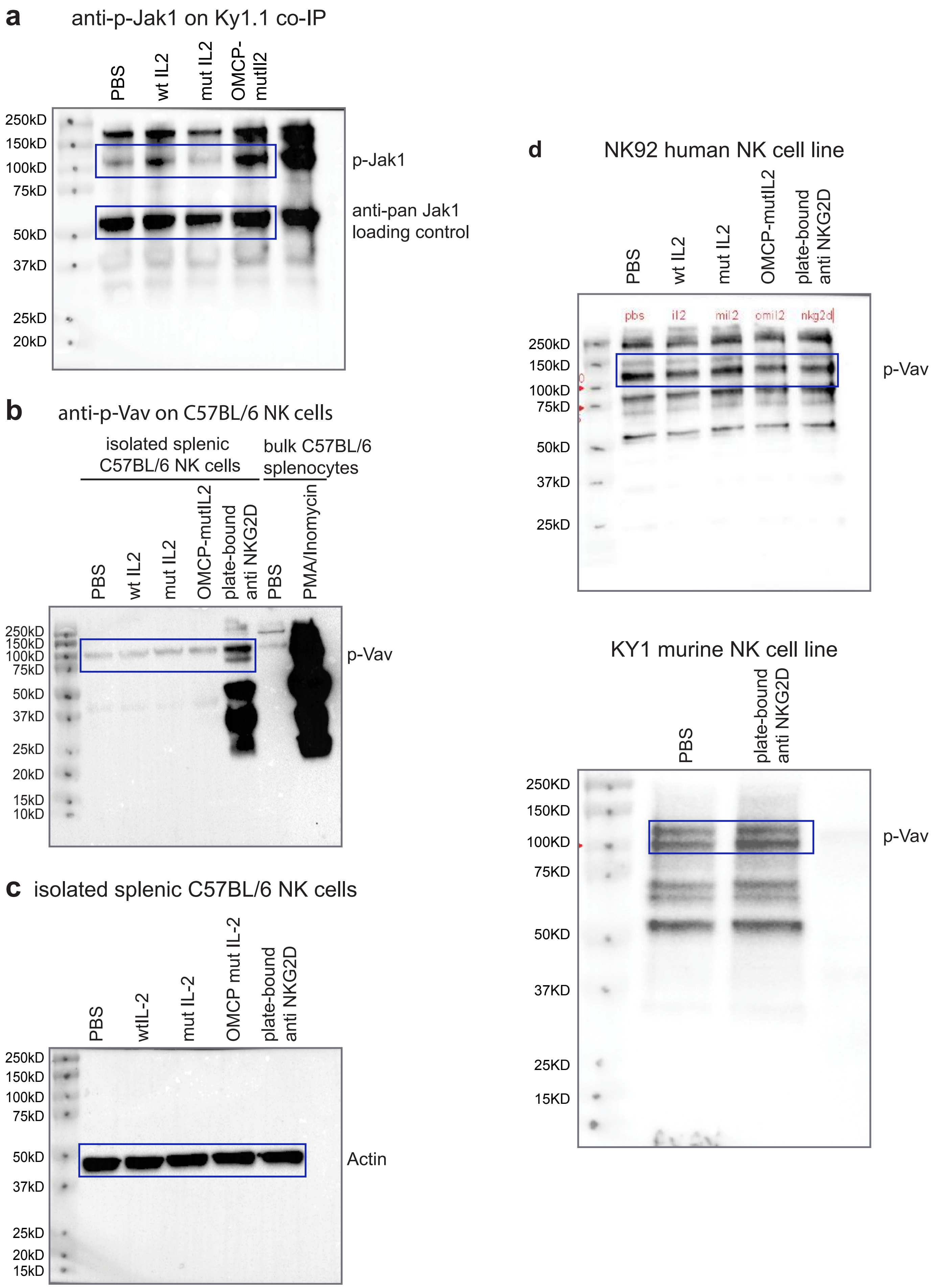

Supplementary Figure 7: Full Western blots. (a) Full Western blot of phospho-Jak1 demonstrated in figure 6c. (b) Full Western blot of phospho-Vav as demonstrated in Figure 6d. (c) Actin control for Figure 6c. (d) Full Western blots of phospho Vav on NK92 (top) and Ky1.1 (bottom).
